# A Central Small Amino Acid in the VAMP2 Transmembrane Domain Regulates the Fusion Pore in Exocytosis

**DOI:** 10.1038/s41598-017-03013-3

**Published:** 2017-06-06

**Authors:** Benoît Hastoy, Pier A. Scotti, Alexandra Milochau, Zahia Fezoua-Boubegtiten, Jorge Rodas, Rémi Megret, Bernard Desbat, Michel Laguerre, Sabine Castano, David Perrais, Patrik Rorsman, Reiko Oda, Jochen Lang

**Affiliations:** 1 Laboratory of Membrane Chemistry and Biology (CBMN), UMR CNRS 5248, Université de Bordeaux, Allée de Geoffroy St Hilaire, 33600 Pessac, France; 2 Université de Bordeaux, 351 Cours de la Libération, 33400 Talence, France; 3 Oxford Centre for Diabetes, Endocrinology and Metabolism, University of Oxford, Churchill Hospital, Oxford, OX3 7LJ UK; 4 Laboratoire de l’Intégration du Matériau au Système, UMR CNRS 5218, 351 Cours de la Libération, 33400 Talence, France; 5 Institut Polytechnique de Bordeaux, Avernue des Facultés, 33405 Talence, France; 6 Interdisciplinary Institute for Neuroscience, UMR CNRS 5287, 146, rue Léo-Saignat, 33077 Bordeaux, France

## Abstract

Exocytosis depends on cytosolic domains of SNARE proteins but the function of the transmembrane domains (TMDs) in membrane fusion remains controversial. The TMD of the SNARE protein synaptobrevin2/VAMP2 contains two highly conserved small amino acids, G_100_ and C_103_, in its central portion. Substituting G_100_ and/or C_103_ with the β-branched amino acid valine impairs the structural flexibility of the TMD in terms of α-helix/β-sheet transitions in model membranes (measured by infrared reflection-absorption or evanescent wave spectroscopy) during increase in protein/lipid ratios, a parameter expected to be altered by recruitment of SNAREs at fusion sites. This structural change is accompanied by reduced membrane fluidity (measured by infrared ellipsometry). The G_100_V/C_103_V mutation nearly abolishes depolarization-evoked exocytosis (measured by membrane capacitance) and hormone secretion (measured biochemically). Single-vesicle optical (by TIRF microscopy) and biophysical measurements of ATP release indicate that G_100_V/C_103_V retards initial fusion-pore opening, hinders its expansion and leads to premature closure in most instances. We conclude that the TMD of VAMP2 plays a critical role in membrane fusion and that the structural mobility provided by the central small amino acids is crucial for exocytosis by influencing the molecular re-arrangements of the lipid membrane that are necessary for fusion pore opening and expansion.

## Introduction

The release of neurotransmitters and hormones occurs by regulated exocytosis which consists of the fusion of the membrane of vesicles with the plasma membrane^1^. The SNARE proteins, a set of evolutionarily conserved proteins, form the core machinery for membrane fusion. During exocytosis, the cytosolic domains of the plasmalemmal proteins SNAP25 and syntaxin-1 interact with the vesicular protein VAMP2 to form the SNARE complex. The SNARE complex folds from the N- to C-terminal as a zipper, which brings the two adjacent membranes together. The resulting mechanical stress is believed to be transduced into membrane fusion via the juxtamembrane domain^2^. The molecular basis of membrane fusion and the associated lipid rearrangement remain poorly understood. It involves a series of intermediate steps such as stalk formation, hemifusion (fusion of adjacent membrane layers) prior to fusion pore opening and expansion^3^. These intermediate steps also imply changes in membrane tension and pressure as well as in local SNARE protein concentration^4, 5^.

Whereas the link between structure and function of the soluble cytosolic SNARE domains is well understood, much less is known about the role of the transmembrane domains (TMDs). Previous studies on potential function of the TMDs in fusion have provided contradictory results. The TMD of VAMP2 or homologues could be replaced by long-chain lipids or unrelated transmembrane domains^6–9^ and single point mutations did not affect exocytosis^10^, although some changes in dimerization were observed *in vivo* ^11^. In addition, replacement of the VAMP2 TMD by a lipid-anchored domain was found to be without effect on exocytosis when measurements were made at a supraphysiological (8 mM) extracellular Ca^2+^ concentration^12^. However, exocytosis was reduced by 80% at a more physiological Ca^2+^ concentration (2 mM). The latter observations raise the interesting possibility that the TMDs may play a role in exocytosis that extends beyond being merely a membrane anchor. This is further supported by a recent work from Dhara and colleagues who replaced half of VAMP2 TMD and showed altered fusion pore kinetics^10^.

The concept that the VAMP2 TMD influences exocytosis is suggested by two pieces of evidence: (1) Synthetic peptide containing alternating leucines and valines as well as helix-breaking residues recapitulate the physico-chemical properties of VAMP2 TMD and are fusogenic in model bilayers^13–16^; and (2) although membrane lipid mixing persists in reconstituted and cellular systems after replacement of the TMD with lipids or unrelated transmembrane protein sequences, the opening and lifetime of the fusion pore opening is strongly reduced^17–20^.

VAMP TMDs exhibit α-helical and β-sheet conformation in model membranes depending on the peptide/lipid ratio^15, 21^, a parameter that increases during membrane fusion^4, 22, 23^. This structural flexibility correlates with an increased fusogenicity^13–15^. The VAMP TMDs are capable of both vertical^24^ and lateral movements^21, 25, 26^ within the lipid membrane. Such movements require local peptide unfolding/refolding and may result in perturbation of the lipid reorganization that precedes fusion pore formation. These observations suggest a potential role of the TMD in membrane fusion but the precise mechanisms involved remain unknown. Understanding of fusion pore regulation is of fundamental importance and may also reveal the molecular basis of human diseases^27^.

## Results

### Conserved small amino acids in VAMP2 transmembrane domain

**Figure 1:**
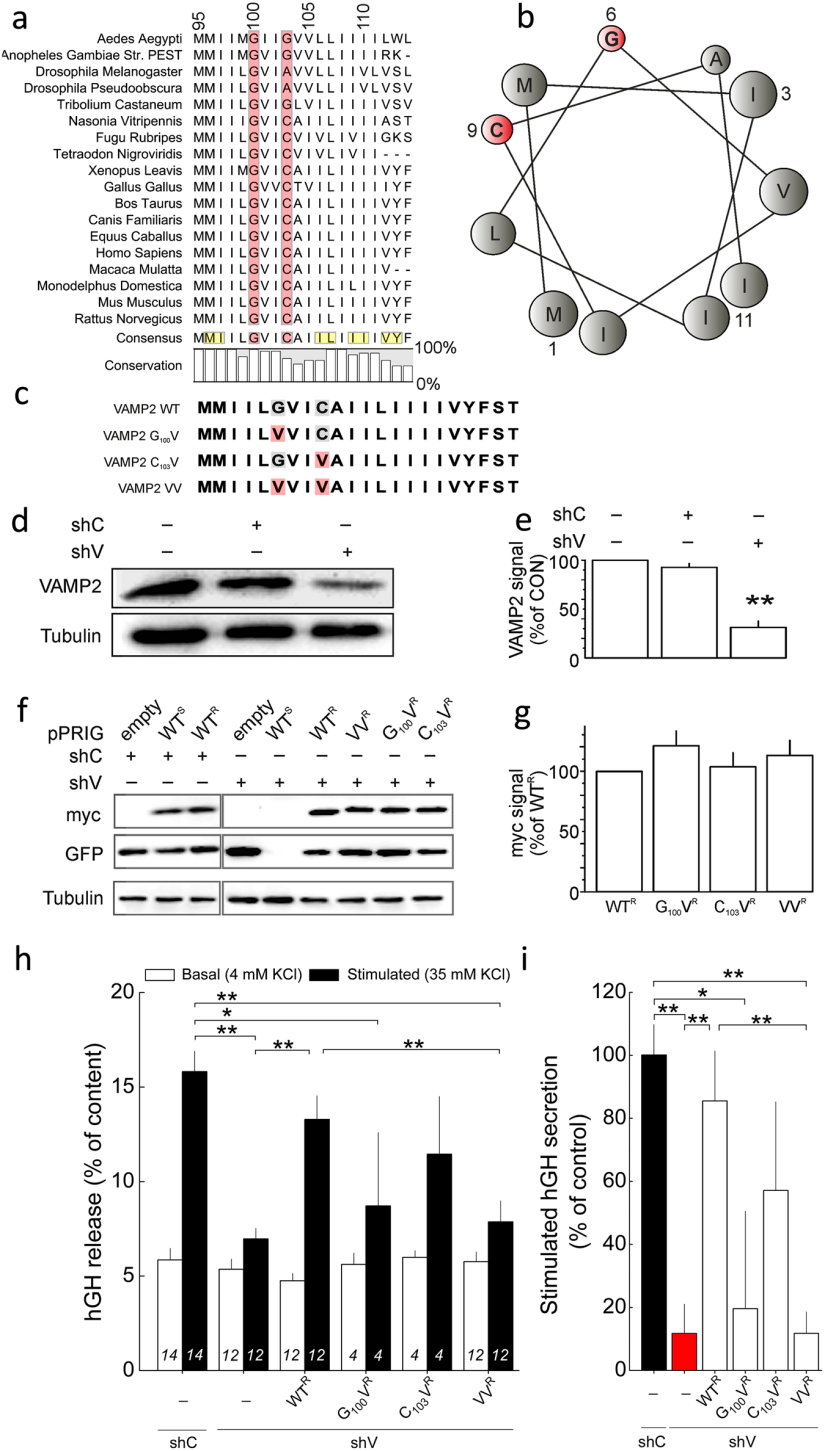
Conserved small residues in the N-terminal portion of the transmembrane domain of VAMP2 have a role in exocytosis. (**a**) Phylogenetic comparison of the VAMP2 transmembrane domain. The highly conserved small amino acids at the position 100 and 103 are highlighted in red. In the consensus sequence, residues that localize on the same aspect of the helical structure as G_100_ and C_103_ are presented in yellow. (**b**) Helical wheel presentation of the N-terminal half of the transmembrane domain highlighting the clustering of small (red) to medium-sized residues on a single face. (**c**) Sequences of wild-type and mutant TMDs; point mutations are highlighted in red. Mutations of small residues in the transmembrane domain (TMD) inhibit secretion in PC12 cells. Effects on hormone secretion were determined for mutants of the VAMP2 TMD after knock-down of endogenous VAMP2. (**d**) Transient expression of shRNA against VAMP2 (shV) but not control shRNA (shC) reduces expression of VAMP2 in PC12 cells. Tubulin immunoreactivity is shown for comparison. Full blots are given in Supplemental Fig. 8. (**e**) Quantification of silencing efficiency of shC and shV on endogenous VAMP2 as indicated. Data have been normalised to control conditions (no transfection). N = 3, **p < 0.01. (**f**) Re-expression of shRNA-resistant VAMP2. Cells were co-transfected with a plasmid bearing shC or shV, a bicistronic plasmid expressing either eGFP alone (pPRIG empty) or both eGFP and VAMP2. Wild-type VAMP2 was either sensitive (WT^S^) or resistant (WT^R^) to shV. All mutants were resistant to shV (VAMP2^R^). Full blots are given in Supplemental Fig. 8. (**g**) Quantification of re-expression of exogenous VAMP2^R^s normalised to control (WT^R^). N = 3. (**h**) Reconstitution of depolarization-induced secretion. PC12 cells were transiently co-transfected with a plasmid expressing the human growth hormone (hGH), shC or shV, and the indicated VAMP2^R^ (WT^R^ or mutant in the TMD). Basal and stimulated hGH secretion are given. Mean values ± S.E.M., N as indicated. *p < 0.05, **p < 0.01 (ANOVA and Bonferroni). (**i**) Same data as g presented as percentage of reconstitution of stimulated secretion. Statistics as in h.

To define targets for mutagenesis, we compared VAMP2’s TMD across several species (Fig. 1a). VAMP2 TMD is dominated by repetition of hydrophobic residues such as leucine (L), or β-branched hydrophobic isoleucine (I) and valine (V). Phylogenetic comparison reveals the conserved presence of a tiny amino acid such as glycine (G), in VAMP2 at position 100. G is followed three residues away by a second tiny residue which is either a glycine (G), cysteine (C) or an alanine (A). The helical wheel projection of the consensus sequence (MMIILGVICAIILIIIIVYF, Fig. 1a) indicates that these residues localize to the same side of the α-helix (Fig. 1b) and are framed by isoleucines, leucines and valine/tyrosine (Fig. 1a, yellow). The high degree of conservation of these residues points towards functional importance^16^. Indeed, *in silico* molecular dynamics and structural data suggest that they produce a kink in protein that decouples the N-terminal and C-terminal halves of the TMD^28, 29^. Thus, mutations that reduce backbone mobility at this position are predicted to affect hormone secretion. To this end, we mutated G_100_ and C_103_ into valines, singly or in combination (G_100_V, C_103_V and G_100_V/C_103_V (VV)) (Fig. 1c). From a structural dynamics perspective, this β-branched amino acid should make the carbon backbone more rigid and in turn reduce mobility^30^.

### Mutations in VAMP2 TMD reduce hormone secretion

The mutants were first tested in rat pheochromocytoma PC12-cells, a widely used model to study regulated exocytosis. Here, the expression of endogenous VAMP2 was suppressed using an shRNA specific for VAMP2 (shV; Fig. 1d; full blots see Supplementary Data) and was compared to a control shRNA (shC). ShV down-regulates endogenous VAMP2 expression by nearly 70% whereas shC was without effect (Fig. 1e). We attribute remaining VAMP2 to non-transfected PC12-cells (see Methods). We next expressed exogenous VAMP2 (mutant and wild-type [WT]) rendered resistant to shV (indicated by an ^R^) by introducing six silent mutations. The resulting constructs are referred to as WT^R^, G_100_V^R^, C_103_V^R^, VV^R^ (Fig. 1f). These exogenous shV-resistant forms of VAMP2 were expressed concomitantly from a single plasmid with a separate GFP (to detect transfected cells). Exogenous VAMP2s contained also an N-terminal myc-tag. The expression of the exogenous WT VAMP2, either sensitive (WT^S^) or resistant (WT^R^) to shV, is not modified by the co-transfection with shC (Fig. 1f). When WT^S^ was co-transfected with shV, its expression was abolished as was the expression of GFP (both WT^S^ and GFP are translated from the same mRNA). By contrast, when the shV-resistant constructs were co-expressed with shV, both VAMP2 and GFP expression was unaffected (Fig. 1f). The expression of the different exogenous VAMP2s was similar for all constructs (Fig. 1f,g). For all experiments, the reporter used to measure functional consequences of such mutations is co-transfected which excludes the interference of non-transfected cells in regard to the interpretation of the data (see Methods).

We next examined the subcellular localization of the different shV-resistant VAMP2 constructs using the same transfection protocol as for functional assay. Cells were co-transfected with plasmids expressing VAMP2 constructs of interest, the shV and the neuropeptide Y (NPY) fused to Red Fluorescent Protein (RFP, i.e. NPY-RFP) as a marker for the Large Dense Core Vesicles (LDCVs) (Suppl. Fig. S1). VAMP2 WT^R^ as well as the mutants co-localized with NPY-RFP, indicating correct targeting to the secretory granules (Suppl. Fig. S1).

To analyze the functional consequences of the VAMP2 mutations in the TMD, we explored the capacity to restore hormone secretion in cells in which endogenous VAMP2 had been down-regulated. For this assay, the reporter used is the human growth hormone (hGH), which is co-secreted from the LDCV (Fig. 1h,i) but not expressed in non-transfected cells. Secretion was stimulated by membrane depolarization ([K^+^]_o_ 35 mM for 10 min), thereby activating voltage-gated Ca^2+^ channels. Depolarization increased hGH release almost 3-fold above basal (Fig. 1h). Whereas down-regulation of endogenous VAMP2 strongly reduced [K^+^]_o_-stimulated secretion, expression of WT^R^ rescued depolarization-evoked secretion to levels not different from control cells. Expression of G_100_V^R^ and C_103_V^R^ only partially restored [K^+^]_o_-induced secretion and the VV^R^ mutant was unable to support depolarization-evoked exocytosis altogether. For display, the stimulated hGH secretion normalized to the control condition (shC) is summarized in Fig. 1i. These data suggest that mutations in the TMD of VAMP2 have strong functional consequences and that this applies especially to G_100_ (the most conserved residue, Fig. 1a).

### The small residues in the transmembrane domain are required for structural dynamics

**Figure 2:**
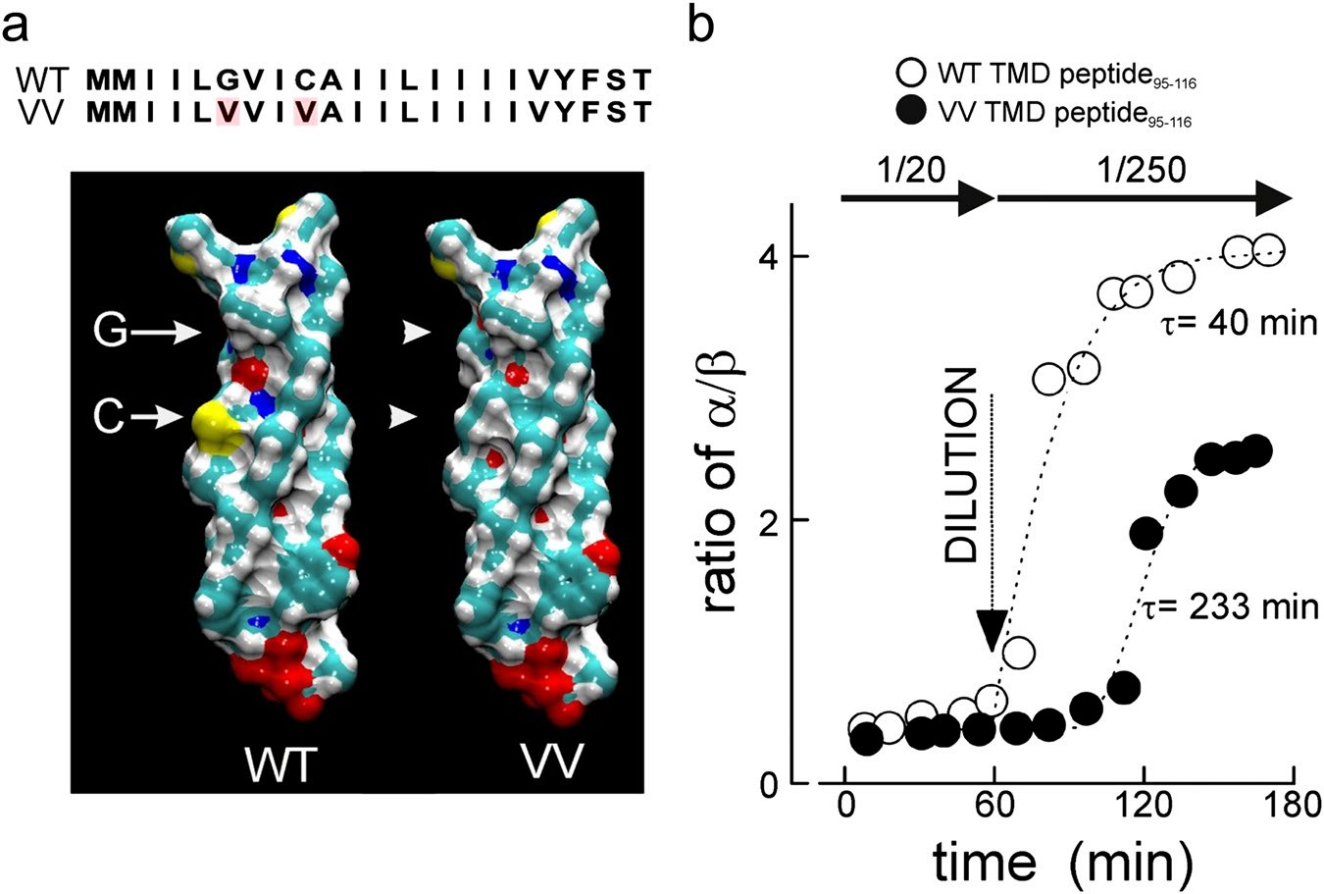
The VV mutation reduces structural flexibility of the VAMP2 TMD. (**a**) Sequences and space-filling models of the transmembrane domain of the WT and VV mutant VAMP2 as an α-helix. Yellow designates sulphur, green carbon, white hydrogen and red oxygen. Arrows designate positions of G_100_ and C_103_, arrowheads point to the volume changes in the mutated sites. (**b**) ATR–IR spectra of the synthetic peptide VAMP2_95-116_ (WT or VV mutant) in a lipid multi-bilayer (DOPC) were obtained at a peptide/lipid ratio of 1/20 at room temperature. After 1 h peptides were diluted with DOPC to a peptide/lipid ratio of 1/250 and structural changes measured for 2 h at room temperature. The ratios of α-helices vs β-sheets are given for wild-type (○) or the VV mutant (●). The curves were obtained fitting exponential growth. The time constants (τ) are given next to the curves.

Figure 2a, shows the predicted 3D α-helical structures of wild-type and mutant TMDs. The tiny amino acid G_100_ indents the α-helix (arrow in Fig. 2a) and may provide flexibility. The substitution of G_100_ by V tends to fill this indentation (Fig. 2a, VV, upper arrowhead). By contrast, substitution of only the second small amino acid, C_103_, by valine has less dramatic effects on the 3D organization (Fig. 2a, VV, lower arrowhead). If the indentation that results from G_100_ is functionally important, then mutations should affect the structural behavior of VAMP2 TMD and the β-branched valine should rigidify the carbon backbone^30^.

To evaluate the effect of mutations on conformation and structural dynamics, we employed physicochemical approaches that allow dynamic studies of conformational changes and protein-lipid interactions in model membranes. We first tested structural mobility of the TMD alone using synthetic peptides (VAMP2_95-116_) and focused on the VAMP2 VV mutant as it had the most pronounced biological effect (see Fig. 1h). Attenuated Total Reflectance-Fourier Transformed Infra-Red spectroscopy (ATR-FTIR) enables quantitative measurements of the protein structure within lipid bilayer membranes. For display, data are expressed as the ratio between α helices and β sheets. The peptides were mixed with lipids at an initially fixed ratio of 1:20 (Fig. 2b) and at this ratio both WT and VV-TMDs are mainly present as β sheets with an α/β ratio of ~0.4. However, when the peptide/lipid ratio is lowered to 1:250 by addition of lipids, the α/β-ratio increased ten-fold with a time constant (τ) of 40 min reflecting now mainly α-helical conformation. Similar dynamics were previously observed for VAMP1^21, 26^. The time constant is slower than those generally measured in reconstituted fusion assays but the latter are conducted at 35 °C^20, 31^ and not at 22 °C as here. The conformational changes in the VV-TMD peptide were considerably smaller (α/β-ratio: 2.5) and occurred with ~6-fold slower kinetics (τ = 233 min) as compared to the wild- type upon lowering of the peptide/lipid ratio.

**Figure 3:**
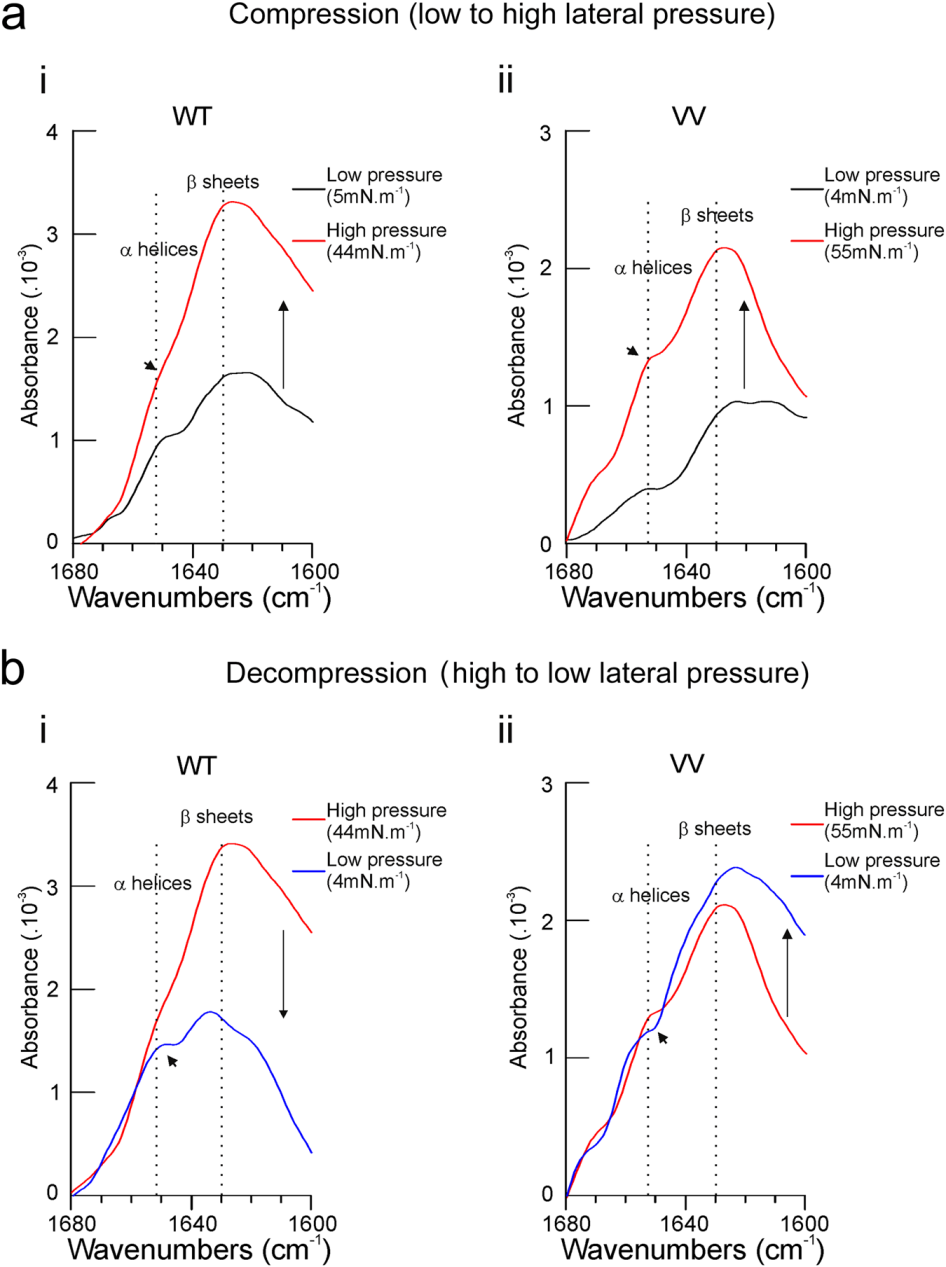
The VV mutations in VAMP2 TMD reduce structural dynamics. Structural features of VAMP2 WT or VAMP2 VV full-length proteins were measured by infrared spectroscopy (PMIRRAS) in a Langmuir through at different lateral pressure. Note that increases in pressure increase local protein concentrations and peptide/lipid ratios. All experiments were performed at room temperature. Full-length proteins were embedded in DMPC monolayer at the protein/lipid ratio of 1/50 (Comparable results were obtained at peptide/lipid ratios of 1/20, data not shown). α-helical conformations and β-sheets conformations were detected at 1653 cm^−1^ and 1630 cm^−1^, respectively. (**a**) Structural behaviour of either VAMP2 WT or VV-TMDs during compression of the DMPC monolayer (a*i* and a*ii*, respectively). (**a** *i*) Starting from a low lateral pressure (5 mN.m^−1^, black curve), a shoulder at 1653 cm^−1^ (corresponding to α helices) was detected. During compression to 44 mN.m^−1^, this shoulder becomes less prominent (red curve, arrowhead). (**a** *ii*) Identical procedure performed with VV from 4 mN.m^−1^ (black curve) to 55 mN.m^−1^ (red curve). Note the persistence of the shoulder (arrowhead) at high pressure. (**b**) Structural behaviour of either VAMP2 WT or VV-TMDs during decompression of the DMPC monolayer (b*i* and b*ii*, respectively). (**b** *i*) To facilitate comparison, the high pressure curve from A has been superimposed (red traces). Decompression of the monolayer from 44 to 4 mN.m^−1^ restores the α helix shoulder (b*i*, blue curve, arrowhead), whereas decompression from 55 mN.m^−1^ to 4 mN.m^−1^ on membrane containing VV does not change the absorbance curve (**b** *ii*, blue curve and arrowhead).

These observations suggest that the VV mutant has a reduced capacity to undergo conformational changes. We extended these observations using polarization modulation infrared reflection-adsorption spectroscopy (PMIRRAS) (Fig. 3). This provides qualitative information on the conformational dynamics of the TMD and allows the experimental control and variation of variables such as pressure that translates also into protein concentrations^32^ both expected to vary during membrane fusion^4, 5, 22, 23^. Since artificial bilayer formation is unstable and often results in triple layers, this technique employs lipid monolayers, but offers the possibility to use full-length protein. The proteins are embedded in a model membrane, which is exposed to variable lateral pressures to mimic the effects of varying the local concentration of VAMP2 in the membrane and of the physical stress known to be important during membrane during fusion^5^. The PMIRRAS spectra of the WT TMD at low lateral pressure (Fig. 3a*i*, black curve) showed a peak at 1630 cm^−1^ and a shoulder at 1653 cm^−1^, indicating a mixture of α-helices and β-sheets. Compression of the lipid layer containing WT protein increased absorbance (reflecting increased local protein concentration), which was particularly pronounced at 1630 cm^−1^, indicating an increase β-sheet configuration (red curve). These observations using the full-length protein confirmed the measurement obtained with the peptide using ATR and bilayers. These changes were fully reversible upon decompression (Fig. 3b*i*).

We repeated these measurements with the VAMP2 VV mutant (Fig. 3a*ii–*b*ii*). As for the WT, compression increased the local concentration (increased absorbance) but had minor effects on the shape of the spectrum. The peaks at 1653 cm^−1^ and 1630 cm^−1^ increased to the same extent, indicating that in contrast to the WT transition from α-helical into β-sheet organization does not occur (Fig. 3a*ii*). Unlike what was seen for the WT protein, the absorbance did not decrease upon decompression (Fig. 3b*ii*). This indicates that the mutant VAMP2 has a reduced ability to undergo a conformational change. Note also that absorbance remains elevated after pressure reduction suggesting that the VV protein-lipid monolayer (unlike the one containing WT) does no longer relax. The intermediate mutants (G_100_V and C_103_V) were also analyzed (Supplementary Fig. 2) and also exhibited a tendency to decreased conformational changes.

Collectively, these data indicate that protein/lipid ratios strongly influence secondary structure on VAMP2 TMD and in a reversible manner, analogous to what we reported for VAMP1^21, 26^. The sensitivity to its environment is a remarkable physical property of VAMP and it is easy to see how both the pressure and protein concentration can vary at the fusion site and thereby modify TMD dynamics^5^.

### The small residues in the transmembrane domain affect membrane fluidity

**Figure 4:**
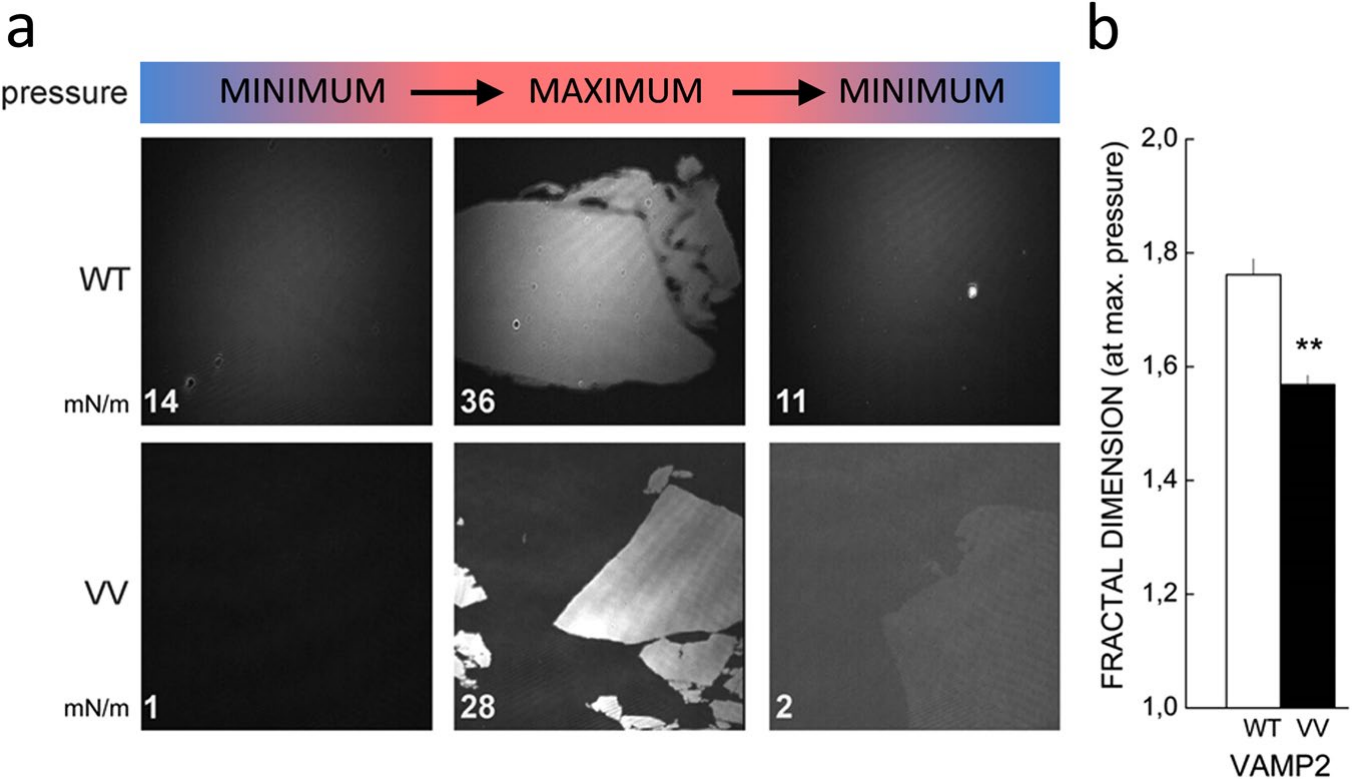
The VV mutation in VAMP2 TMD modifies the fluidity of the membrane. Viscosity of model membranes were imaged by ellipsometry in a Langmuir through using DMPC membranes and either VAMP2 WT or VAMP2 VV full-length recombinant protein. (**a**) Representative images from DMPC model membranes mixed with the indicated mutant (1/50 nominal peptide/lipid ratio) obtained by ellipsometry in a Langmuir trough. Images were taken at initial low lateral pressure (left panel), at maximal lateral pressure (middle panel) and after relaxation (to low pressure, right panel). Measured lateral pressures are given at the bottom left corner of each image (mN/m). For VAMP2 WT, during the increase of the lateral pressure, the DMPC membrane evolves from homogenous monolayer to a monolayer bearing distinctive domains of different thickness (clear and dark zones). At the maximal pressure (36 mN/m), the patterns (round shapes, no sharp angles) indicate regions of great fluidity. By decompressing the system, the membrane returns fully to its original homogeneity. By contrast, an increase of the lateral pressure on membrane containing VV leads to the formation of ‘jagged’ patches with many sharp angles, a mark of membrane rigidity (28 mN/m). These changes persist upon decompression. (**b**) Quantification of fractional dimension of images obtained in ellipsometry by using the box counting dimension (mean D_B_, a logarithmic factor which varies between 1 and 2). Mean ± S.E.M., n = 6; **2p < 0.01 (t-test).

Exocytosis ultimately depends on the reorganization of lipid membranes at the fusion site. In PMIRRAS, persistence of elevated absorbance upon decompression of VV-mutant containing membranes suggests that the mutations modify the relaxation of the membrane after physical stress such as lateral pressure (see Fig. 3b*ii*). We investigated the impact of VAMP TMDs on membrane fluidity using infrared ellipsometry. This optical and contact-free method enables characterization of material properties such as viscosity. We imaged membranes seeded with the different VAMP2 proteins by the same compression/decompression cycle as used for PMIRRAS (Fig. 4). At low lateral pressure (left column), the membrane is of homogenous thickness (as indicted by uniform grey colour). As pressure increases (middle column), brighter areas appear against a darker background. The shape of these bright areas provides information on the fluidity/rigidity of the lipid membrane: round shapes indicate great fluidity, whereas jagged shapes indicate rigidity (‘crystalline’ properties). For intermediate viscosities, elongated shapes without sharp angles are detected. Compression resulted in the appearance of bright areas for the WT and all mutant VAMP2s. Whereas the shapes of these domains are rounded/elongated for WT, shard-like structures are seen for VV mutants. Upon decompression, these changes were fully reversible for the WT but less so for the membrane containing VAMP2 VV. Quantitative analysis of images (Fig. 4b) demonstrates a decrease in fractal dimension for the VV mutant, reflecting the observed loss in meandering patterns. Membranes where intermediate mutants were embedded present also clearly a specific behavior (Supplementary Fig. 3). Membranes bearing C_103_V presented similar patterns and capacity to relax the system. On the other hand, shard-like structures appeared during compression of G_100_V mutant with incomplete ‘relaxation’ upon decompression, echoing the observations of VAMP2 VV.

*In silico* simulations indicate that the TMD configuration influences the flexibility of the juxtamembrane cytosolic linker and the SNARE motif^33^.We examined by immunoprecipitation whether mutant VAMP2 is able to form SNARE complexes and whether the dimerization properties of TMDs^34^ are altered (Supplementary Fig. 3). Within the limits of the assay used, we did not observe any differences between WT and VV mutant VAMP2, suggesting that both variants are equally likely to form SNARE complexes. Moreover, the VV mutant did not have an increased tendency to form dimers.

### Effects of VAMP2 TMD mutant on exocytosis measured by membrane capacitance

**Figure 5:**
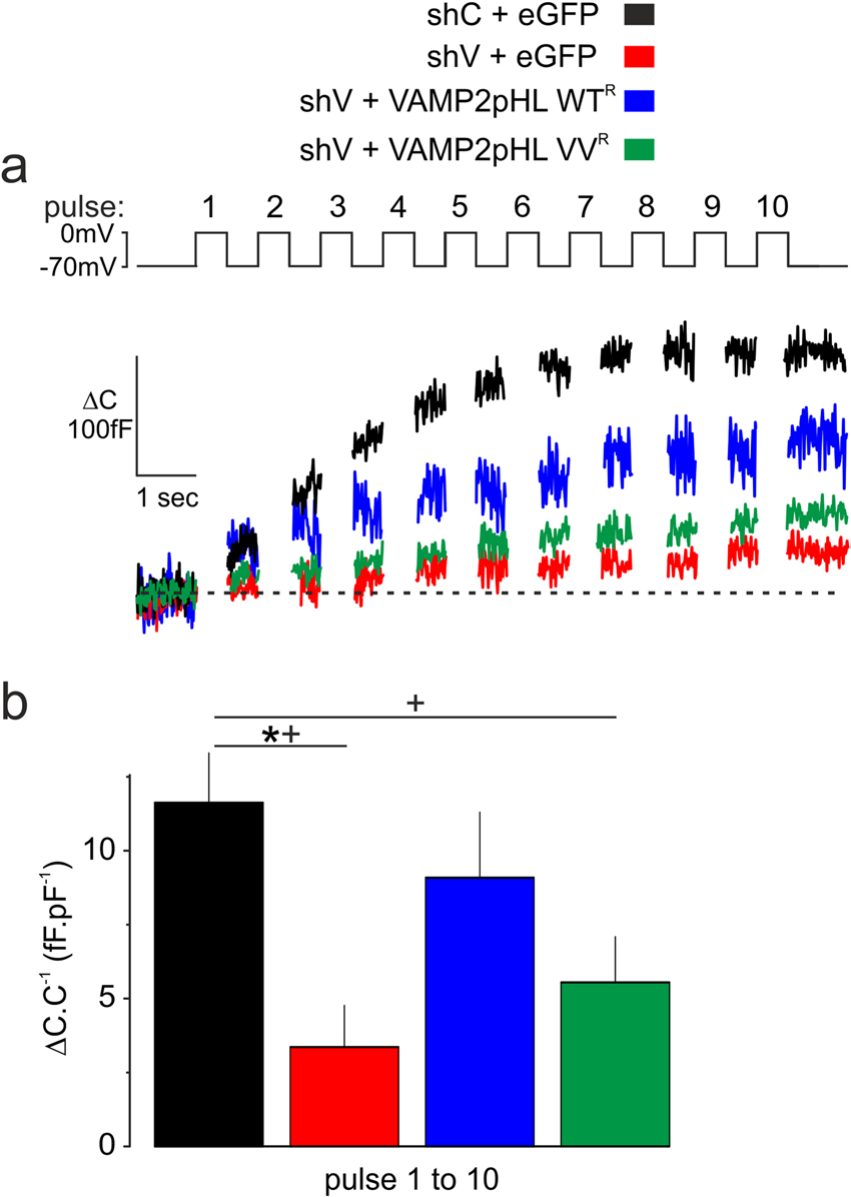
Effects of mutant VAMP2 TMDs on exocytosis measured by membrane capacitance in INS-1 832/13 clonal β-cells. Reconstitution of exocytosis by VAMP2 WT or VAMP2 VV and kinetics of vesicle pools were measured in INS-1 832/13 clonal β-cells expressing VAMP2pHL WT^R^ or VAMP2pHL VV^R^ after knock-down of endogenous VAMP2. (**a**) Representative traces of cumulative increase in membrane capacitance (ΔC in fF) elicited by 10 depolarizations (top panel) from −70 mV to 0 mV applied at 1 Hz. Cells were co-transfected with either shC + eGFP (n = 5, black trace), shV + eGFP (n = 6, red trace), shV + VAMP2pHL WT^R^ (n = 6, blue trace) or shV + VAMP2pHL VV^R^ (n = 10, green trace). (**b**) Quantification of the cumulative increase of capacitance normalized to cell capacitance (ΔC.C^−1^) at the end of the train. Although presenting a clear trend, the difference in the cumulative exocytosis measured in cells expressing either VAMP2pHL WT^R^ or VAMP2pHL VV^R^ did not reach statistical significance (ANOVA and Tukey, p = 0.093, *p < 0.05). However, differences became significantly evident when comparing the kinetics of exocytosis for WT^R^ or VV^R^ to those of the reference control shC (ANOVA and Dunnett, +, p < 0.05).

The structural data show that G_100_ had a great effect on VAMP2 TMD dynamics, which not only affected the TMD itself but also the behavior of the surrounding lipid membrane. The double mutant (G_100_V/C_103_V, VV) had the strongest effect on secretion and structural dynamics. The impact of this TMD mutant on depolarization-evoked secretion was explored by capacitance measurements of exocytosis (Fig. 5) in the clonal β-cell line INS-1 832/13 (in the following referred to as INS1-cells), a model of Ca^2+^-dependent secretion^35, 36^.

Similar to PC12-cells, endogenous VAMP2 expression was specifically knocked-down. The different constructs were expressed at comparable levels and colocalized with insulin in fixed cells or NPY-RFP in living cells (Supplementary Fig. 5a,b). Expression of the constructs was not associated with compensatory up-regulation of VAMP3 (Supplementary Fig. 5a), unlike what has previously been reported in chromaffin cells^37^. No expression of VAMP1 was detected in INS1-cells or non-differentiated PC12-cells as reported previously^38, 39^ (Supplementary Fig. 4D). It was ascertained that only some 4% of endogenous VAMP2 remain in co-transfected cells (Supplementary Fig. 6). Collectively, these considerations argue that responses observed reflect the behavior of the exogenously expressed VAMP2.

In control INS1-cells (transfected with shC and eGFP, included for the identification of successfully transfected cells), a train of ten 500-ms depolarizations from −70 mV to 0 mV (top panel, Fig. 5a) evoked a biphasic response with an initial large step increase in membrane capacitance, followed by progressively smaller increases (Fig. 5a, representative black trace). For analysis, changes in cell capacitance (ΔC) have been normalized to the initial cell capacitance to compensate for variations of cell size (i.e. ΔC.C^−1^, Fig. 5b,c). Silencing VAMP or expressing the mutants had no impact on cell size (not shown). The total increase in cell capacitance during the train is shown in Fig. 5b. After transfection with shV (again with eGFP), depolarization-evoked exocytosis was nearly abolished (Fig. 5a,b, red trace and column) but almost fully rescued in cells expressing WT^R^ (Fig. 5a,b, blue trace and column), whereas VV^R^ was ineffective (Fig. 5a,b, green trace and column), at least during the 10-s stimulation period used here. The inhibition of exocytosis cannot be attributed to reduced Ca^2+^ channel activity (Supplementary Fig. 7).

### Effects of VAMP2 TMD mutant measured by TIRF imaging of exocytosis

**Figure 6:**
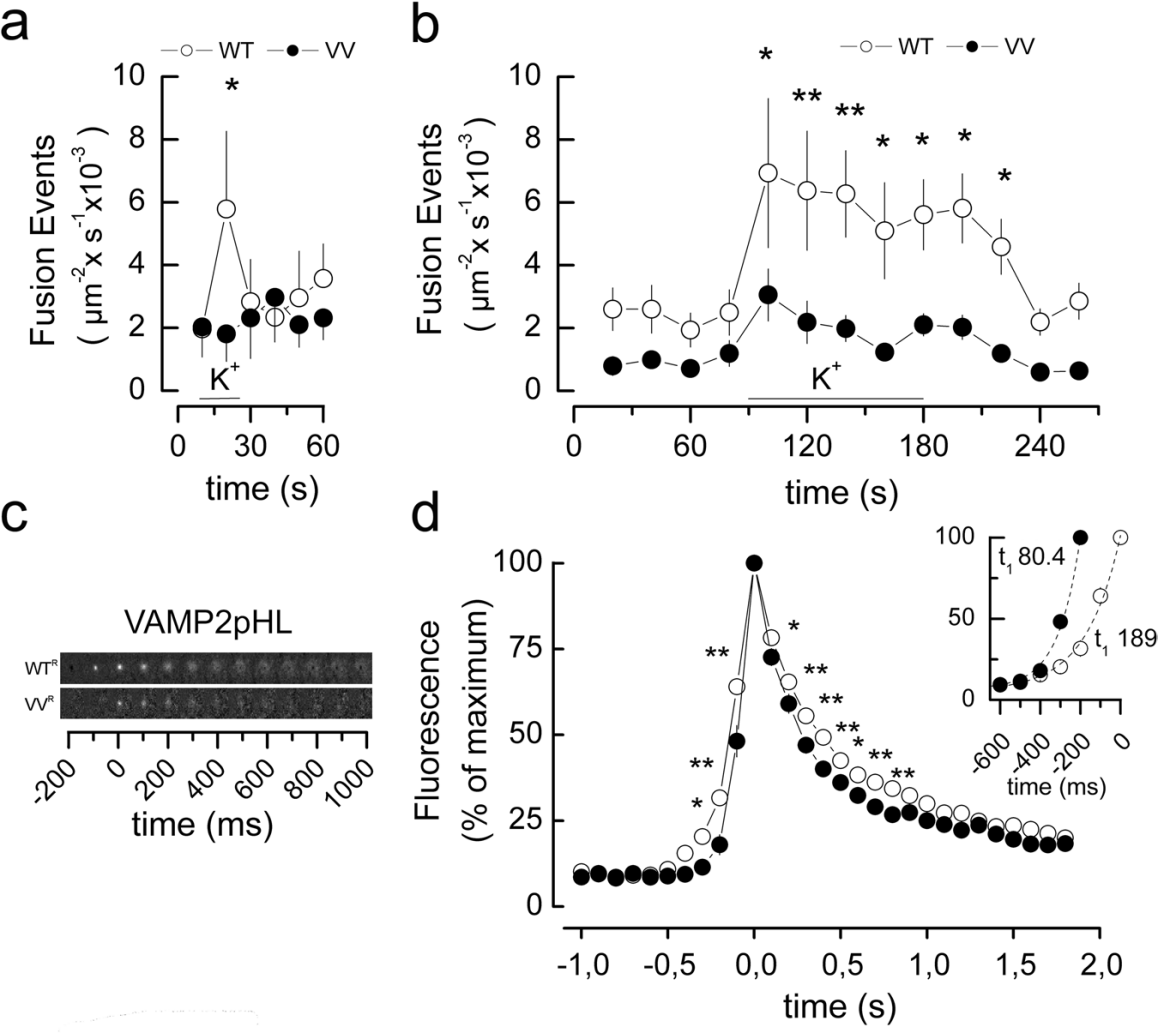
The VV mutation in the TMD of VAMP2 alters exocytosis and fusion pore kinetics. Exocytosis and release kinetics were determined by near-field TIRF microscopy in PC12 and in INS-1 832/13 clonal β-cells expressing VAMP2 WT or the VAMP2 VV mutant after knock-down of endogenous VAMP2. (**a**) PC12 cells were transiently co-transfected with plasmids encoding VAMP2-pHluorin (WT^R^ or VV^R^) and shRNA directed against VAMP2. Fusion events were recorded by TIRF microscopy and sum of fusion events per 10 s is given for cells stimulated by 90 mM [K^+^]_o_ (WT, n = 6 cells, 237 events; VV n = 4 cells, 139 events total) *2p < 0.05 (Student’s *t*-test). (**b**) Same as in (**a**) but measurements performed on INS-1 832/13. Sum of fusion event per 20 s in transfected cells were stimulated by 35 mM [K^+^]_o_ for 30 s (WT n = 9 cells, 1114 events total; VV n = 9 cells, 476 events total. *2p < 0.05; **2p < 0.01 (Student’s *t*-test). (**c**) Representative films of fusion events in INS-1 832/13 cells. The events are temporally synchronized to the frame of their maximum fluorescence. (**d**) Mean changes in normalized fluorescence immediately before and after exocytosis in INS-1 832/13 cells expressing either VAMP2pHL WT^R^ (open circles) or VV^R^ (closed circles). For display the fluorescent mean events were synchronized to their maximum fluorescence. Note narrower peak for VV as compared to WT. *2p < 0.05; **2p < 0.01 VAMP2 WT vs. VV (Student’s *t*-test). INSERT: Events synchronized for first event with a mean >10% of maximal fluorescence and curves fitted for first order exponential growth. Note shorter time constant τ for VV (closed circles) as compared to WT (open circles). The time constants τ_up_ for the fluorescence increases averaged 189 ± 28 ms for WT^R^ and 80 ± 7 ms for VV^R^ (p < 0.05; F-test). Similarly, the decay of fluorescence following the peak was slower for WT^R^ (time constant τ_down_: 558 ± 57 ms) than for VV^R^ (τ_down_: 315 ± 17 ms) (p < 0.05; F-test).

We corroborated the observations on hormone secretion and capacitance measurements by total internal reflection microscopy (TIRF) to monitor release of docked granules^40^. To visualize vesicles undergoing fusion, we used VAMP2 WT^R^ or VV^R^ coupled with the pH-sensitive eGFP-pHluorin (VAMP2pHL; same as used in Fig. 5). For this probe fluorescence increases when the vesicular pH neutralizes during opening of the fusion pore^41^. Both measurements were performed in the presence of shV in PC12 and INS-1 832/13 cell lines (Fig. 6a,b). In PC12-cells expressing WT^R^, membrane depolarization (induced by 90 mM [K^+^]_o_) evoked a transient stimulation of exocytosis. Consistent with the hormone secretion measurements in Fig. 1h, expression of VV^R^ instead of WT^R^ abolished this stimulation whilst not affecting basal release. When the same type of experiment was conducted in INS1-cells expressing WT^R^, high-[K^+^]_o_ (35 mM) depolarization resulted in a sustained stimulation of secretion. Stimulated secretion was strongly inhibited in cells expressing VV^R^. Basal secretion before elevation of [K^+^]_o_ tended to be reduced in VV^R^-expressing cells but this effect did not attain statistical significance. Figure 6c shows TIRF images of representative fusion events recorded at different time points in cells expressing either WT^R^ or VV^R^. We analyzed the kinetics of the fusion events and Fig. 6d shows averaged fluorescence increases associated with LDCV exocytosis. Whereas the increase to maximal fluorescence took ≈500 ms for VAMP2-WT vesicles, the delay was only 300 ms for the VV^R^ vesicles resulting in distinct time constants and the decay of fluorescence following the peak was likewise slower for WT^R^ than for VV^R^. In summary, the TIRF measurements using membrane-bound VAMP2pHL as probe indicate that the number of release events is much reduced in VV^R^ cells and that exocytosis (once initiated) proceeds either more rapidly or the life-time of the fusion pore is reduced.

### Effects of VAMP2 TMD mutant on the kinetics of the fusion pore

**Figure 7:**
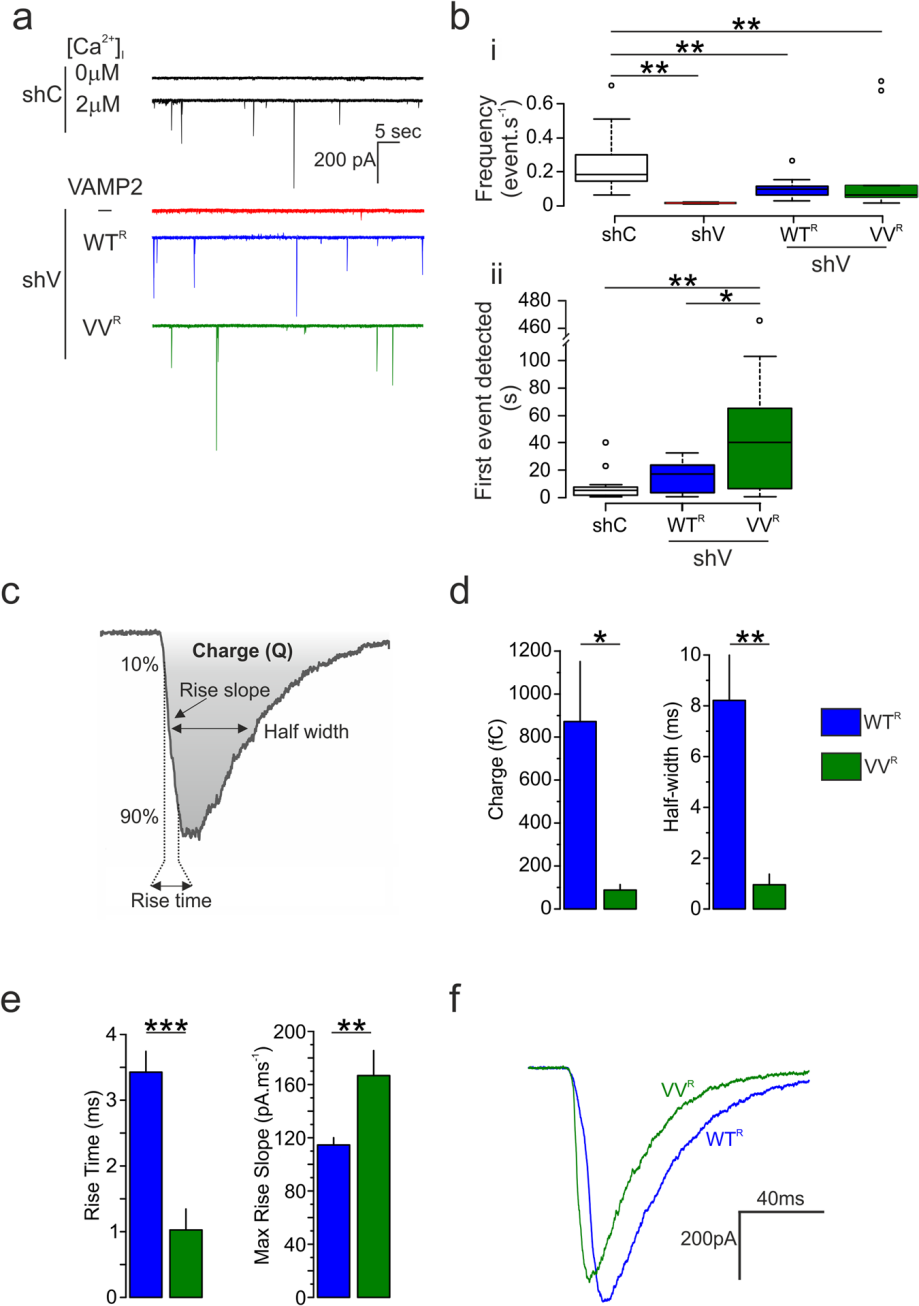
VAMP2 G_100_/C_103_ influence fusion pore opening. Measurements of quantal release of ATP via P2X_2_ receptor mediated currents from INS-1 832/13 cells expressing VAMP2 WT (G_100_/C_103_) or VV (G_100_V/C_103_V) after knock-down of endogenous VAMP2. (**a**) Currents triggered by ATP release. Top traces show INS-1 832/13 cells transfected with shC (black trace) and infused with media containing 0 or 2 μM free calcium. The 3 bottom traces are from cells co-transfected with plasmid P2X_2_-YFP, shC or shV, and VAMP2-pHL (WT^R^ or VV^R^), respectively (black, shC; red, shV + empty vector; blue, shV + VAMP2pHL WT^R^; green; shV + VAMP2pHL VV^R^). (**b** *i*) Frequency of events per second (ANOVA and Tukey shC vs. shV p = 0.004, vs WT^R^ p = 0.004, vs. VV^R^ p = 0.001; shC, 12 cells, n = 802 events total; shV, 6 cells, n = 30 events total; WT^R^ 12 cells, n = 388 events; VV^R^, 8 cells, n = 695 events; _°_, outliers). (**b**) Response delay defined as time required for the first event to be detected (ANOVA and Tukey, shC = 4 ± 0.86 s, WT^R^ = 14, 43 ± 3.21 s, VV^R^ = 36.90 ± 11.48 s. (**c**) Schematic illustration of the charge, the 10% to 90% rise time, the rise slope, and the half width of the current generated by P2X_2_R. These parameters are measured for the fusion pore kinetics study. (**d**,**e**) Mean values of the medians for the charge, the half width (**d**) and rise time and its slope (**e**), respectively (*p < 0.05, **p < 0.01, t test). (**f**) Superimposition of currents of similar amplitude elicited from cells expressing VAMP2pHL WT^R^ (blue) and VV^R^ (green).

The TIRF measurements indicate that VV^R^ influences the mechanics of fusion pore expansion. We used INS1-cells transfected with the ATP-sensitive P2X_2_ receptor (P2X_2_R) to monitor kinetics of single-vesicle exocytosis at a higher resolution^27, 42, 43^. No exocytotic events were observed when cells were infused with a Ca^2+^-free buffer (10 mM EGTA alone; Fig. 7a, grey trace). However, when the cells were infused with buffers containing 2 μM [Ca^2+^]_i_ (mixture of 9 mM Ca^2+^/10 mM EGTA), exocytosis was observed (Fig. 7a, black trace). Knockdown of endogenous VAMP2 abolished Ca^2+^-induced exocytosis (Fig. 7a,b). Exocytosis in shV-treated cells was partially restored when VAMP2WT^R^ or VAMP2VV^R^ was expressed (Fig. 7a,b). No difference in the frequency was observed between the WT^R^ and VV^R^ constructs. (Fig. 7b*i*). However, the first fusion event was delayed by 20 s when cells expressed VAMP2VV^R^ (Fig. 7b*ii*). We quantified the charge and the half-width as well as the rise time and the maximum slope of activation (illustrated schematically in Fig. 7c). Values shown represent averages of the median for each cell. Charge and half-width were reduced by 85% in cells expressing the VAMP2 VV construct (Fig. 7d). The rise time was reduced by 70% and the maximum slope increased by 25% (Fig. 7e). Assuming that the reduction of charge does not reflect a reduction of vesicular ATP content, we hypothesize that VAMP2VV is unable to keep the fusion pore open and promotes kiss and run events rather than full fusion. Figure 7f shows representative WT and VV events with similar peak amplitudes. There were no significant differences between WT and shC events (not shown) for any of the parameters above. Thus, overexpression of VAMP construct *per se* does not affect fusion.

## Discussion

We combined a structural analysis of VAMP2 transmembrane domain dynamics and a characterization of its potential cellular function. Biochemical, electrophysiological and optical approaches demonstrate that VAMP2 modified in the TMD region (G_100_V/C_103_V) is unable to restore exocytosis. Fine analysis revealed a reduced structural mobility of the TMD, an enhanced viscosity of the membranes as well as a delayed but more rapid fusion pore expansion. Even though we observed the most pronounced effect when both G_100_ and C_103_ were mutated into V, the single G_100_V mutation alone was able to alter the membrane viscosity and partially reduced the hormone release. This observation makes us hypothesize that the function of VAMP2 TMD during exocytosis is principally mediated by G_100_.

The critical role of G_100_ has not previously been detected in cellular system. This we attribute to two factors. First, in some studies, the glycine was replaced by residues that should not increase the structural rigidity of the TMD^10, 44, 45^. Second, toxins were used in some studies to inhibit the exocytosis supported by the endogenous VAMP2 but in such systems remaining endogenous TMD may dimerize and compensate for the effects of the studied mutations^8, 11^. Concomitant mutation of C_103_ to β-branched V further increased functional effects. We do not think that this could be explained by changes in intramembrane cysteine palmitoylation^46^ as a mutation of Cys to Leu does not alter the efficacy of VAMP2^10^.

The profound and reversible structural transition of the native TMD (from α-helices to β-sheets) is a unique property of VAMP1^21, 26^ and VAMP2 (current data). Previously the presence of β-sheets has only been observed for synthetic sequences or truncated VAMP2^15, 29^. Our structural analysis was able to capture such dynamics for both the full-length protein and the corresponding TMD peptide. These findings argue that the structural dynamics represents a key property of the transmembrane domain.

Predicting or modeling the structural consequences of mutations is notoriously difficult^47^. The existence of a β-sheet conformation of the TMDs has been detected by previous spectroscopic approaches^15, 21, 26^, even in highly dehydrated membranes^29^ and its functional significance was perhaps not always appreciated because of the lack of dynamic measurements. The failure of *in silico* molecular simulations to predict a β-sheet conformation can be attributed to the constraints of parameter values. The dynamic measurements presented here suggest that the TMDs not only possess great structural flexibility, they also (via the changes in their structure) influence membrane viscosity, a property that easily can be envisaged to influence membrane fusion.

**Figure 8:**
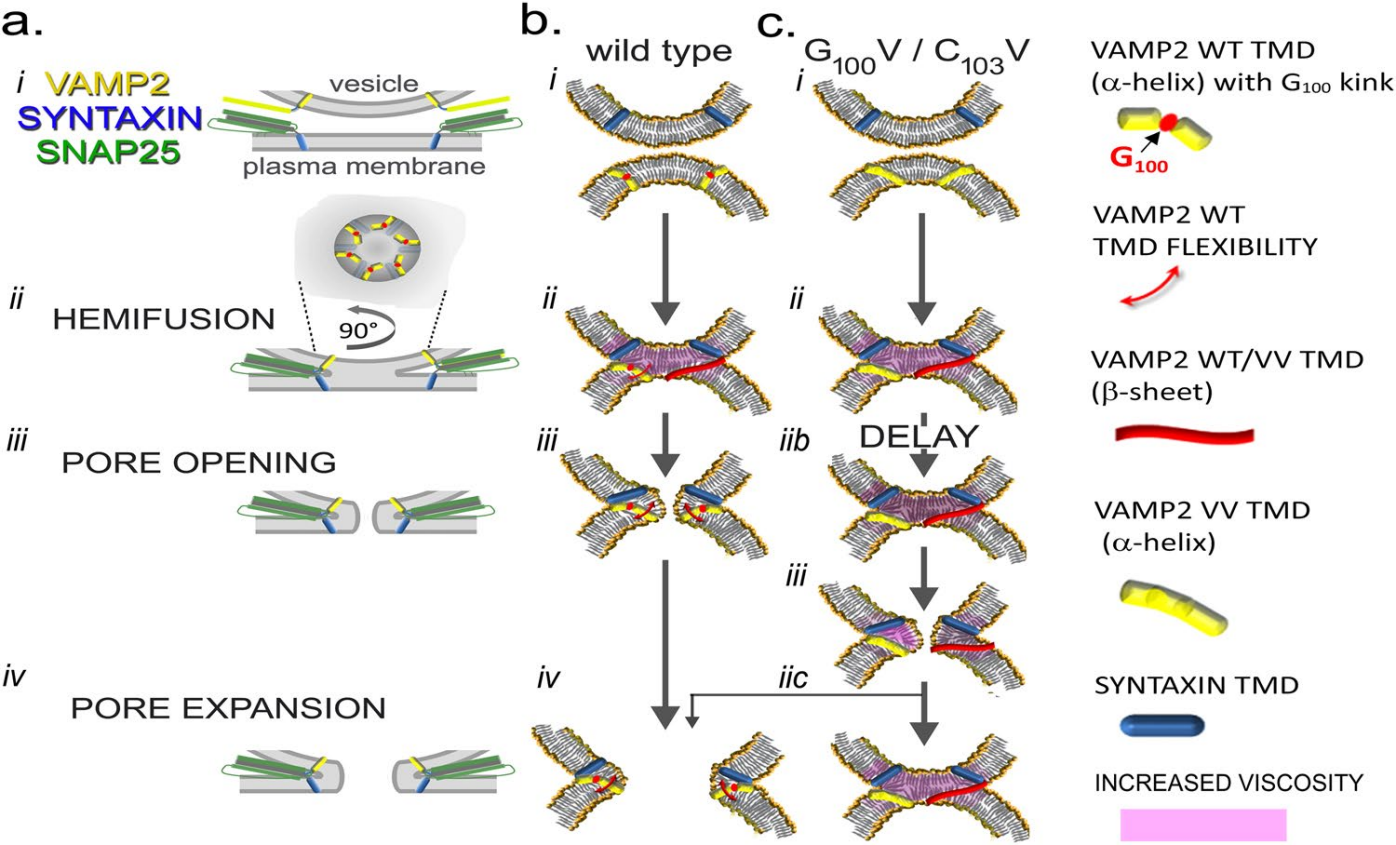
Proposed model of conformation of VAMP2 transmembrane domain during exocytosis. The different steps of exocytosis are viewed from general scale with the presence of the SNARE cytosolic domain (**a**) and more focused on the TMD structure from VAMP2 WT (**b**) and VAMP2 VV (G_100_V/C_103_V) (**c**). Before vesicle docking, VAMP2 is uniformly distributed over the vesicle, corresponding to a low local concentration of VAMP2, and the TMDs are mainly in α-helical conformation (**a** *i*,**b** *i*, **c** *i*). Following docking, VAMP2 proteins concentrate at the fusion site (**a** *ii*). The increased local TMD concentration alters the peptide/lipid ratio and induces the switch of its conformation from an α-helical to a β-sheet conformation. The structural change is accompanied by a tilt of angle of the transmembrane domain from 30° to 54°^21^, promoting the lipid reorganisation and an increase of membrane viscosity (**b** *ii*,**c** *ii*). Once fusion has occurred and following lipid rearrangement, the SNAREs are localised on the same membrane (**a** *iii*). The decrease of the local concentration of VAMP2 and membrane tension results in the return to an α-helical conformation in the case of VAMP2 WT. This is accompanied by an increase of fluidity of the membrane that promotes the opening of the fusion pore for VAMP2 WT (**b** *iii*) and its subsequent expansion (**a** *iv* and **b** *iv*). The VV mutant has reduced capacity for reversible structural changes and is either lacks flexibility around G_100_ or is locked in the β-sheet conformation. This causes a delay in the fusion pore opening in the case of VAMP2 VV (**c** *iib*, **c** *iii*). The persisting increase in membrane viscosity does not permit further expansion of the pore and induces its premature closure in most cases (**c** *iic*). In some cases full fusion may occur as documented by recording of membrane capacitance.

Several observations point towards the relevance of small residues such as the central glycine in TMD structure and function. Model peptides made of leucine-valine repetitions exhibit increased conversion from an α-helical to a β-sheet conformation upon introduction of a central glycine^14^. The significance of TMD leucines and valines is also suggested by the finding that they enhance fusogenicity and are statistically overrepresented in SNARE TMDs and viral fusion sequences^16^. Molecular simulations^28, 33^ and structural data^25, 29^ suggest that the TMD is actually composed of two halves with distinct tilt angles. These two halves are joined by G_100_, which functions as a molecular ‘hinge’. Most recently, it was demonstrated that replacement of either the total TMD or its N-terminal half (including G_100_/C_103_) by polyleucine or poly-isoleucine sequences changes the fusion pore kinetics^10^. Our data indicate that much smaller modifications of the protein (replacing only one or two residues) are sufficient to change structural dynamics along with fusion kinetics and specifically highlight the importance of G_100_ and C_103_ in TMD mobility, membrane viscosity, fusion pore initiation and expansion (Fig. 8). In view of the kink at G_100_ one may speculate whether it is the N- or C-terminal half of the TMD that moves primarily. Indeed, the N-terminal portion is probably rather immobile by its connection to the VAMP juxtamembrane domain interacting with charged cytosolic phospholipid head-groups^48^ and cognate SNARE linkers^49^. In contrast, the C-terminal half is splayed from the TMD of the cognate SNARE protein syntaxin^49^ and cholesterol, known to promote fusion pore opening^50^, has a further effect on the tilt^25^. Addition of charged residues at the C-terminal end restricts VAMP2 TMD mobility and reduces or delays fusion pore opening^24, 51, 52^ which was interpreted as a consequence of reduced vertical “pull” of the TMD into the membrane^53^. However, such a C-terminal modification will also restrict lateral mobility of the transmembrane domain and potentially prevents the tilt of angle accompanying the transition from an α helix to a β sheet conformation^21^.

Based on the findings reported here and taking previous observations into account^54^, we propose a model in which fusion proceeds sequentially via, stalk formation, hemifusion, and fusion pore opening (Fig. 8). The delay and alterations in flux kinetics place the action of G_100_/C_103_ at the level of the initiation of the fusion pore and at its further expansion. The subsequent opening and expansion of the nascent fusion pore constitutes the most-energy-demanding step and requires additional driving forces^3^. Prior to the docking/formation of the SNARE complex, VAMP2 is distributed across the vesicle membrane and the concentration of the transmembrane domains (TMDs) is low, favoring α-helical conformation (Fig. 8a*i*–c*i*). At the initiation of exocytosis, SNARE proteins localize at the fusion site (Fig. 8a*ii*). The resultant increase in the concentration of the TMDs induces a change in peptide/lipid ratios and a conformational change of the VAMP2 TMDs ensues (Fig. 8b*ii*). This may consist of partial helix destabilization and enhanced conformational mobility around G_100_ or - potentially, as extreme outcome –full transition from α-helices to β-sheets. We postulate that such conformational mobility is required to overcome the complex geometry and viscosity of lipid assembly during the transition from hemifusion to fusion pore opening (Fig. 8a*iii*–b*iii*) and may promote lipid mixing. This step is delayed in the case of VAMP2 VV, which remains locked in its conformation and modifies the surrounding membrane properties (Fig. 8c*ii*–c*iib*). Once the fusion pore opened, it expands further in the case of VAMP2 VV (Fig. 8a*iv* and b*iv*). In contrast, in case of VAMP2 VV increased membrane viscosity and tension hinders further expansion and lead to premature closure of the pore in most instances (Fig. 8c*iic*) although full fusion may still occur in some instances as suggested by capacitance measurements (Fig. 5).

The molecular nature of the fusion pore is intensely debated^55^ and has variably been postulated to be either lipidic^56, 57^ or proteinaceous^58, 59^. It is implicit from the model we propose that the fusion pore is – at least partially – of lipidic nature. Regardless of the final outcome of this debate, our findings show an intimate interaction between the dynamics of the VAMP2 transmembrane domains via the central glycine and the fluidity of the lipid membrane. In turn, this interaction influences greatly the likelihood and speed of fusion pore opening and expansion.

## Methods

### Material

1,2-Dimyristoyl-sn-glycero-3-phophatidylcholine (DMPC, CAS 8194-24-6) and 1,2-dioleoyl-sn-Glycero-3-Phosphocholine (DOPC, CAS 4235-95-4) were purchased from Sigma-Aldrich. INS-1 832/13 cells were generously provided by Drs P Maechler and C.B. Wollheim (Geneva, CH)^36^. PC12 cells were obtained from ATCC and used for up to six passages. The following primary antibodies were used: polyclonal or monoclonal anti-myc (Sigma Aldrich, Saint-Quentin, France and Millipore, Molsheim, France respectively), monoclonal anti-VAMP2 (clone 69.1, Synaptic Systems, Göttingen, Germany), monoclonal anti-syntaxin (HPC1, Sigma), monoclonal anti-SNAP25 (Sternberger Monoclonals), polyclonal anti-VAMP1 (Santa Cruz, FL118) and anti-tubulin (BD Biosciences, Le Pont-De-Claix, France). The following secondary antibodies were employed: HRP-conjugated secondary antibodies (GE Healthcare), CY3-coupled secondary anti-mouse, antibody, a Texas-red-coupled anti-guinea-pig antibody (Jackson Immunoresearch) and Alexa, 488 anti-rabbit antibody (Molecular Probes). The following antibodies and plasmids were generously donated: polyclonal anti-GFP (kindly provided by M. Rout, Rockefeller University, New York, NY, USA); NPY-mRFP (Dr. W Almers, Portland, USA) and pPRIG (Dr. Martin, Nice, France).

### Molecular Cloning

To generate shRNAs against rat VAMP2, two 64-mer primers were synthetized according to a published sequence^60^, annealed and the synthetic dsDNA with cohesive ends was directly inserted into the vector pSUPER at *Bgl*II/*Hin*dIII sites, generating pSUPERVAMP2 plasmid (primers are listed below). As control shRNA, we used a pSUPERGL2 encoding shRNA against the firefly luciferase GL2. The whole H1 promoter-shGL2 sequence was taken from a lentiviral vector pLVTHMshGL2 (a kind gift from Dr. V. Haurie, University of Bordeaux, France) and substituted into pSUPER (Oligoengine, Seattle, WA, USA). The plasmid pBKCMV-mycVAMP2 was used as a PCR template in order to insert the myc-tagged rat VAMP2 cDNA sequence into the bi-cistronic vector pPRIGp^61^ at *Bam*HI/*Xho*I sites. pPRIGp-mycVAMP2 vector was used to generate all the subsequent point mutations using the QuikChange Site-Directed Mutagenesis Kit (Stratagene, Le Massy, France), starting with the generation of a shRNA resistant version, pPRIGp-mycVAMP2^R^, by introducing 6 silent mutations within the target for the siRNA.

The following primer were used: shVAMP2, sense: 5′GATCCCCGGACCAGAAGCTATCGGAACTTTCAAGAGAAGTTCCGATAGCTTCTGGTCCTTTTTA3′, antisense: 5′AGCTTAAAAAGGACCAGAAGCTATCGGAACTTCTCTTGAAAGTTCCGATAGCTTCTGGTCCGGG5′; VAMP2^R^ wt, sense: 5′GGTCCTGGAGCGGGACCAAAAACTGAGCGAACTGGATGATCGC3′, antisense:5′GCGATCATCCAGTTCGCTCAGTTTTTGGTCCCGCTCCAGGACC3′;VAMP2^R^ G_100_V, sense: 5′GATCATCTTGGTAGTGATTTGCGCC3′, antisense: 5′GGCGCAAATCACTACCAAGATGATC3′; VAMP2^R^ C_103_V, sense: 5′GGGAGTGATTGTCGCCATCATCC3′, antisense: 5′GGATGATGGCGACAATCACTCCC3’; VAMP2^R^ G_100_V/C_103_V, sense: 5′GATCATCTTGGTAGTGATTGTCGCCATCATCC3′, antisense: 5′GGATGATGGCGACAATCACTACCAAGATGATC3′.

For TIRF and capacitance experiments, mycVAMP2R cDNA was also subcloned into pCDNA3 in frame (at its 3′ end) with the encoding sequence for TEVPHL, i.e. TEV protease-specific cleavage site followed by the pHluorin^41^ encoding sequences. The plasmid encodes full-length, non-truncated VAMP2 followed by the protease sensitive linker SMGSGGDYDIPTTENLYFQGELKTTVDAD and the full-length ecliptic phluorin lacking its first amino acid (M). The linker is predicted to form essentially a random coil and potentially an extended sheet for the last six amino acids^61^. Point mutations within the transmembrane domain were introduced using either pPRIGp-mycVAMP2^R^ or pCDNA3-mycVAMP^R^-PHL as template for site-directed mutagenesis. All constructs were verified by sequencing of both strands. For recombinant protein production and purification, the corresponding cDNA VAMP2 sequences (*Eco*RI-*Xho*I fragments) were taken from recombinant pPRIGp vectors and subcloned into pGEX4T2 vector.

### Protein Expression and Purification

Transmembrane domain peptides of 22 amino acid residues (VAMP2_95-116_) were synthesized and purified as previously reported^21^. Recombinant GST-VAMP2 (rat) was produced in *E. coli* BL21 (DE3), solubilized in Triton-X100 containing buffer and bound to glutathione beads^62^. After exchanging the detergent for 0.8% octylglucoside, VAMP was eluted from the beads by thrombin cleavage, concentrated and further purified by FPLC (Superdex75). Purified proteins were stored at −80 °C in phosphate-buffered saline containing 0.8% octylglucoside and 10% glycerol^26^ (Supplementary Fig. 1).

### Cell Culture, Transient Cotransfections and Immunoblots

Cell culture of INS-1 832/13 or PC12 cells and secretion assays were performed as previously described^63^. Cells were periodically checked for mycoplasma and found to be negative. Cells were transfected as published^63^ and cultured for three days before assays. Upon co-transfection of PC12 cells with shV, VAMP2 constructs and NPY, 82 ± 3% of transfected cells co-expressed VAMP2 constructs and NPY, 13% ± 3% only NPY and 5, 3 ± 2% only VAMP2 (n = 8 coverslips). SDS-Page and immunoblots were performed as described^62^. Immunoblots were developed using an ECL system and images were taken (Fluorochem 8000) at different time points to ensure linearity. Signals were quantified using Alfa-Ease FC software (Alpha Innotech) after suppression of local background and normalized to tubulin immunoreactivity from the same immunoblot experiment.

### Immunoprecipitation

INS-1 832/13 cells were harvested 48 h after transfection. Immunoprecipitation of GFP fused to VAMP was realized using GFP-Trap®_MA beads (Chromotek; Martinsried/Germany). Cells were lysed in 250 µl of the following buffer: 20 mM HEPES pH 7.3, 100 mM KCl, 5 mM MgCl2, 0.5 mM EDTA, 1% Triton X-100 and protease inhibitors. After 30 min lysis on ice cells were centrifuged 10 min at 20.000 g. Supernatants (~230 µl) were diluted with 300 µl of lysis buffer without TritonX-100. 20 µl of beads were washed 3 times in the same buffer and 500 µl of diluted extract were applied onto it. After 2 h of head over head incubation at 4 °C, beads were washed 3 times and resuspended in 100 µl of 2X SDS-PAGE buffer.

### Immuno-isolation of Transfected Cells

Immuno-isolation was performed similar as described previously^64^. 72 h hours after transfections INS832-13 cells were harvested and suspended in PBS containing 2 mM EDTA. Aliquots (total fractions) were taken before adding anti-CD8 Dynabeads (Thermofisher, Waltham, MA, USA) equilibrated in PBS-EDTA. Cells and Dynabeads were incubated at 4 °C for 30 min and supernatants were removed by magnetic trapping (yielding the “unbound” fraction). Beads were subsequently washed 3 times with PBS-2 mM EDTA before being suspended in Laemmli sample buffer (providing the “bound” fraction). Total, unbound and bound fractions (2.5%, 2.5% and 50% of each fraction respectively) were analyzed by SDS-PAGE on 12% polyacrylamide gels followed by Western blotting. PVDF membranes were probed with anti-VAMP2, anti β-tubuline and anti-GFP antibodies. VAMP2 expression levels were determined either using a standard curve of purified recombinant VAMP2 or serial dilutions of samples. Images were quantified using Alfa-Ease FC software (Alpha Innotech) after suppression of local background.

### Secretion Assays

PC12 cells were washed twice with glucose containing Krebs-Ringer buffer (KRBG), composed of (mM): 135 NaCl, 3.6 KCl, 3.5 NaHCO, 0.5 NaH_2_PO_4_, 0.5 MgCl_2_, 1.5 CaCl_2_, 25 glucose, 10 HEPES, pH 7.4 with NaOH, supplemented with 0.1% w/v of BSA. Secretion was measured during 10 min either at normal 3.6 mM or elevated 35 mM [K^+^]_o_. When [K^+^]_o_ was increased, [Na^+^] was simultaneously lowered to maintain iso-osmolarity. Note that these K^+^ concentrations are expected to result in lower secretion rates than the concentrations of 48 or 90 mM widely used. Supernatants were centrifuged and human growth hormone (hGH) determined by ELISA (Roche Diagnostics, Meylan, France).

### Transmembrane Domain Comparisons

Among the sequences of VAMP2 collected by Kloeppers *et al*.^65^, those with well delineated transmembrane domains were retained and alignments were performed using Vector NTI 11 (Invitrogen, Life Technologies SAS, Villebon sur Yvette, France).

### Electrophysiology

The electrophysiological recordings were performed using and EPC-10 amplifiers and the Pulse software (version 8.30 or later; Heka Elektronik, Lambrecht, Germany). Exocytosis was detected as changes in cell capacitance, which was estimated by the Lindau-Neher technique implementing the “Sine + DC” feature of the lock-in module^66^. The amplitude of the sine wave was 20 mV and the frequency set as 500 Hz The standard whole-cell configuration was used, and the pipette-filling medium contained (mM) 125 Cs-glutamate, 10 CsCl, 10 NaCl, 1 MgCl2, 0.05 EGTA, 3 Mg-ATP, 0.1 cAMP, and 5 HEPES (pH 7.2 using CsOH). Ten 500 ms depolarizations of from −70 to 0 mV were applied at a frequency of 1 Hz. The responses were measured as the increase in membrane capacitance between the prestimulatory level and the new steady-state value and were normalized by the size of the cell^35, 67^.

### Kinetics of ATP Released Using P2X_2_ Receptor and Current Analysis

Transfected INS1-832/13 cells were patched in whole cell configuration with an intra-pipette buffer containing 115 mM CsCl, 10 mM NaCl, 1 mM MgCl_2_, 5 mM HEPES, 3 mM Mg-ATP, 0 mM (for the control) or 9 mM CaCl_2_ and 10 mM EGTA (calculated free [Ca^2+^]_i_: 0 or 2 μM), pH 7.15 (NaOH). Traces were exported to Clampfit to analyze the rise time (t_10–90%_), and the rise slope of each event. Events with an amplitude between 20 pA and 700 pA were analyzed to avoid interference from the noise background and compound exocytosis.

### Confocal and TIRF Imaging

For immunocytochemistry and imaging by confocal laser microscopy, cells were fixed using 4% paraformaldehyde in PBS and processed as reported^62^, Confocal images were acquired as 8 bit images (308 × 308 pixels, 5.28 µm × 5.28 µm) using a Zeiss LMS 510 Meta confocal laser microscope and a Plan-Apochromat 63x/1.4 OilDIC objective with the following wavelengths and emission filters: λ_ex_ 405 nm, BP420-480; λ_ex_ 488 nm, LP505; λ_ex_ 543 nm, BP560-615; λ_ex_ 633 nm, LP650. Image analysis was performed after thresholding using the plugin Coloc2 for ImageJ^68^.

For TIRF microscopy, transfected cells were observed using an Olympus microscope (IX 71) equipped with TIRF illumination fed with Dual Color Laser (Cobolt, Sweden) (473 and 561 nm wavelengths). Images were taken with a 60 × 1.49 NA TIRF objective. Evanescent field properties were controlled with 0.2 µm TetraSpeck fluorescent microspheres (Invitrogen) before each experiment. Fluorescence emission were filtered using a 525/50 m band-pass filter (Chroma Technology) and images were acquired continuously (100 ms exposure time per frame) with an EMCCD camera (QuantEM, Roper Scientific, France) (1 pixel = 178.9 nm) for at least one minute. INS-1 832/13 Cells were stimulated by the main perfusion of 35 mM KCl KRBG. PC12 cells were stimulated by local perfusion with 90 mM KCl in modified KRBG (containing 50 mM NaCl) using an electrovalve.

For image treatment, the brightest pixel of the fusion event was determined as the spatial and temporal origin of the event using Matlab (Mathworks, Natick, MA, USA). Automatic detection of the fusion event is based on the pattern of VAMP2-pHL fluorescence variations during exocytosis. This is typically composed by an increase in fluorescence intensity for two frames (0.2 s) followed by a decrease over maximally seven subsequent frames (0.7 s). Events were detected using an advanced version of a published protocol^69^ and detected events were confirmed or rejected according to visual inspection. Diffusion analysis has been performed on INS-1 832/13 data after normalization to lowest values = 0 and maxima (fusion) set to 100. Data were fitted using Origin8 Pro (OriginLab Corporation, Northampton, MA, USA) using the concatenate function for fitting of replicates. Fitted datasets were further compared for statistical difference by F-test in the Origin-Pro package.

### Spectroscopy

Monolayer experiments and polarization modulation-infrared reflection-adsorption spectroscopy (PMIRRAS) were performed on a computer-controlled Langmuir film balance (Nima Technology, Coventry, UK) as described previously^26^. All experiments were performed at room temperature (22 °C). Since bilayer formation is unstable and often results in three layers in PMIRRAS, monolayers are used. The signal derived from the TMDs was isolated by deconvolution as described^21, 26^.

The morphology of protein/lipid monolayers at the air-water interface was observed by ellipsometry^26^ using an iElli2000 microscope (NFT, Göttingen, Germany) equipped with a doubled frequency Nd-YAG laser (532 nm, 50 mW), a polarizer, a compensator, an analyzer, and a CCD camera. The lateral resolution of pictures with the x10 magnification lens was about 2 μm. The imaging ellipsometer was used at an incidence angle close to the Brewster angle (54.58°). It operates on the principle of classical null ellipsometry^70^. All experiments were performed at room temperature. Image patterning was determined by fractal analysis using the Fraclac plugin of ImageJ with 12 grid positions^71^. Fractal dimensions given here were obtained by box counting and the values indicated (D_B_) describe the slope of the relationship *ln N*/*ln ε* where N are changes in image details and ε the scale, thus indicating how a pattern’s detail changes with the scale at which it is considered.

To obtain structural models, a structure deposited in the PDB (code 2KOG) was used and mutations were performed using the “mutated residue” facility in the Visual Molecular Dynamics software VMD1.8.6.

### Statistical Analysis and Data Fitting

Data were expressed as the means ± SEM. Differences between two groups were assessed by a two-tailed unpaired Student’s t-test or by a one-way ANOVA using XLSTAT software followed by post-hoc tests as indicated in the legends. The null hypothesis was rejected at the level of p < 0.05.

All data generated or analysed during this study are included in this published article (and its Supplementary Information files).

## Electronic supplementary material

Supplementary Information
