# Supplementary material for: A Central Small Amino Acid in the VAMP2 Transmembrane Domain Regulates the Fusion Pore in Exocytosis
Source: Sci Rep. 2017 Jun 6;7:2835. doi: 10.1038/s41598-017-03013-3 (PMC5460238; doi:10.1038/s41598-017-03013-3)
Supplement: Supplementary file 1 — Supplementary Information [file 41598_2017_3013_MOESM1_ESM.doc]

SUPPLEMENTARY DATA

A Central Small Amino Acid in the VAMP2 Transmembrane Domain
 Regulates the Fusion Pore in Exocytosis

Benoît Hastoy^1,2,3^, Pier A. Scotti^1,2^, Alexandra Milochau^1,2^, Zahia Fezoua-Boubegtiten ^1,2^, Jorge Rodas ^2,4,5^, Rémi Megret^2,4,5^, Bernard Desbat^1,2^, Michel Laguerre^1,2^, Sabine Castano^1,2^, David Perrais^2,6^, Patrik Rorsman^3*^, Reiko Oda^1,2*^, Jochen Lang^1,2*^

^1^ Laboratory of Membrane Chemistry and Biology (CBMN), CNRS UMR 5248, Université de Bordeaux, Allée de Geoffrey St Hilaire, 33600 Pessac, France,

^2^ Université de Bordeaux, 146 rue Léo Saignat, 33077 Bordeaux Cedex, France

^3^ Oxford Centre for Diabetes, Endocrinology and Metabolism, University of Oxford, Churchill Hospital, Oxford OX3 7LJ, UK

^4^ Laboratoire de l’Intégration du Matériau au Système, CNRS UMR 5218,

^5^ Institut Polytechnique de Bordeaux, 351 Cours de la Libération, 33405 Talence cedex, France,

^6^ Interdisciplinary Institute for Neuroscience, CNRS UMR 5297

* Authors contributed equally

**SUPPLEMENTARY FIGURES AND LEGENDS**

**
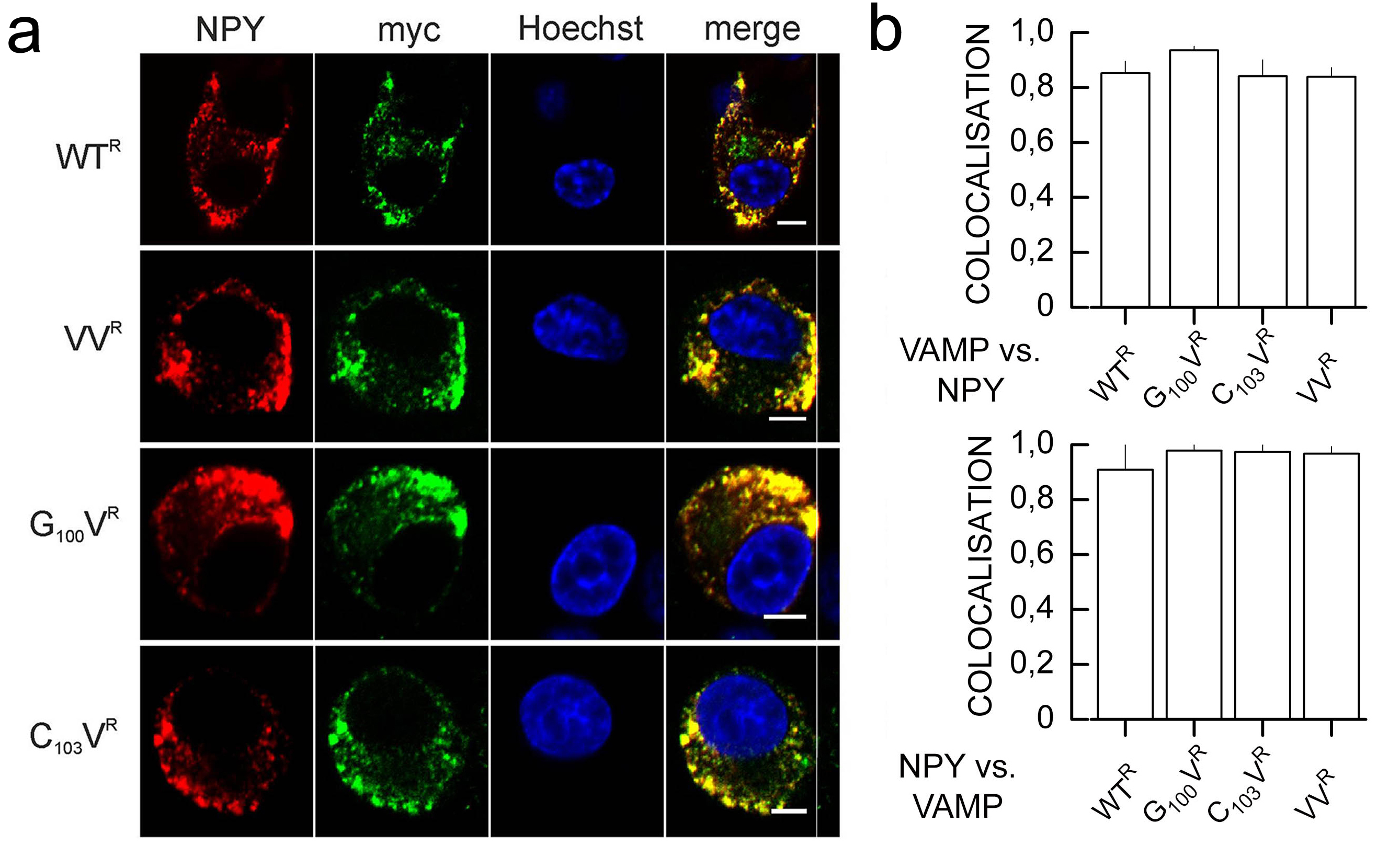
**

Supplementary Figure 1. **Subcellular localisation of VAMP2 transmembrane domain mutants in PC12 cells.** (**a**) Subcellular localisation of transiently expressed WT^R^ or mutant VAMP2^R^ (myc signal) in PC12 cells. Cells were co-transfected with shV, the indicated VAMP2^R^ constructs, and the secretory granule cargo protein NPY. Bars equal 5 µm. (**b**) Determination of colocalization using Mander’s coefficient (N=8).


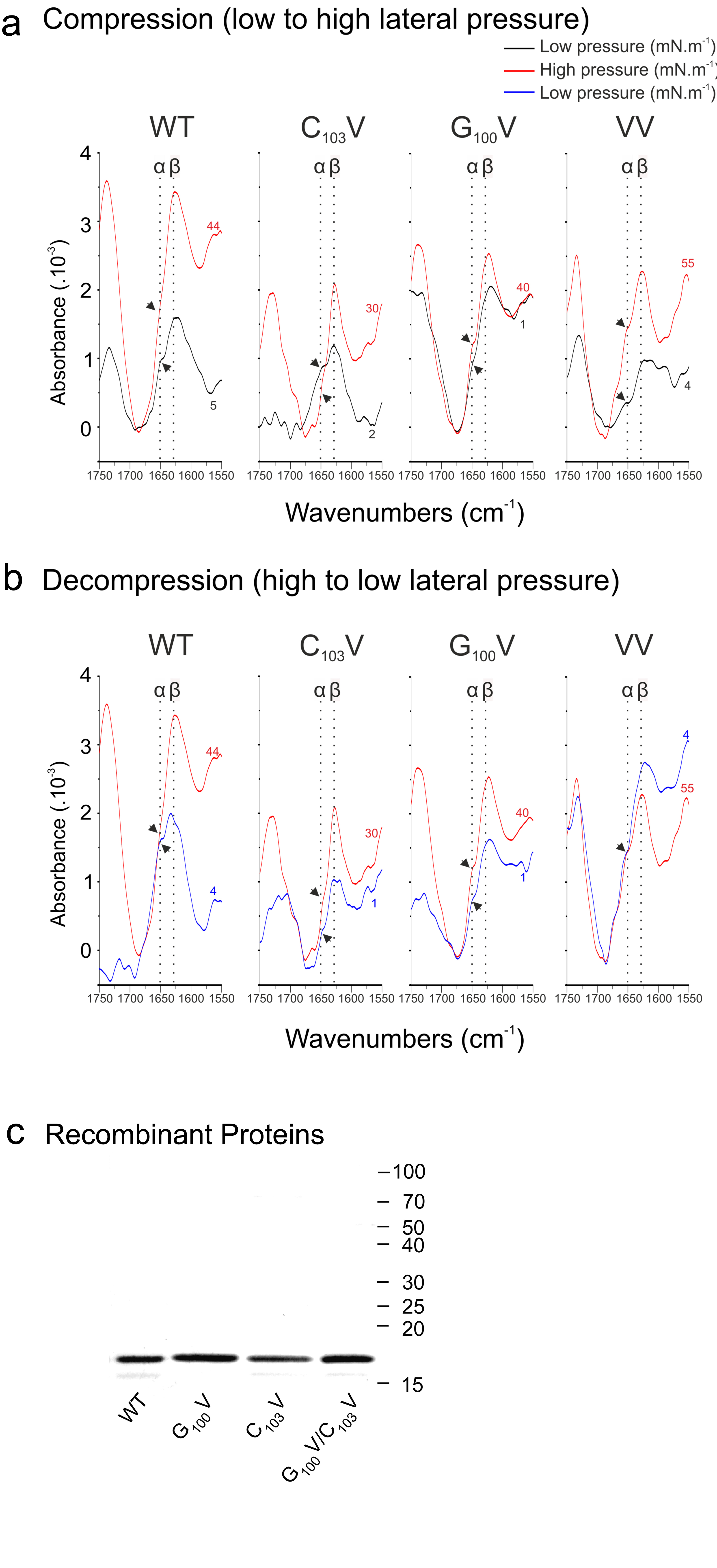


Supplementary Figure 2. **Effect of G _100_V, C_103_V and VV mutations on the TMD structural dynamics.** The overall structure of the transmembrane domain of full-length protein is assessed in DMPC monolayers (protein/lipid ratio 1/20) by PMIRRAS. **a** and **b**: As in Figure 4, α-helical and β-sheet structures are detectable at 1653 and 1630 cm^-1^, respectively. Starting from a low lateral pressure (indicated in mN/m at the right side of each absorbance curve; black curve), membranes were compressed (red curve) and subsequently decompressed (blue curve) as indicated by arrows (right hand side of the panel). Traces represent VAMP2 WT, G_100_V, C_103_V and VV as indicated. Whereas C103V behaved like the WT protein with a decreasing α-helix shoulder at 1653 cm^-1^ at high pressure, G100V behaved more like the VV mutant with a persistent β-peak at 1630 cm^-1^. These differences echo the impact of the mutants on secretion (Fig. 2F). **c**, Recombinant proteins used in spectrometry. Indicated proteins (0.5 μg/lane) were separated by SDS-PAGE and stained with Coomassie Blue. Molecular weight standards are given in kDa.

**
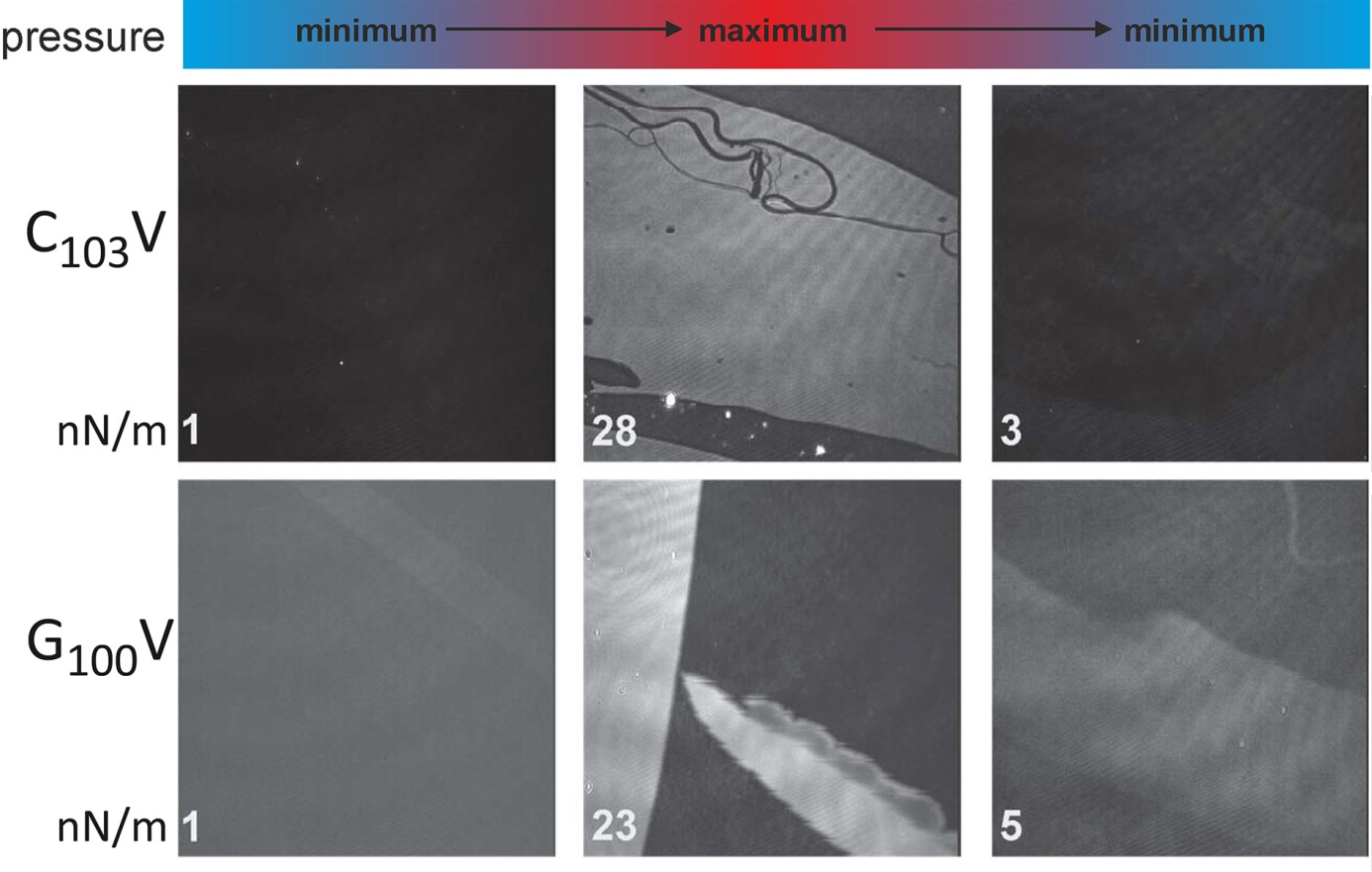
**

Supplementary Figure 3. **Effect of single mutations in G_100_ or in C_103_ in VAMP2 TMD on membrane fluidity.** Representative images from DMPC model membranes mixed with the indicated mutant (1/20 nominal peptide/lipid ratio) were obtained at initial low lateral pressure (left panel), at maximal lateral pressure (middle panel) and after relaxation (to low pressure, right panel). Measured lateral pressures are given at the bottom left corner of each image (mN/m). For VAMP2 C_103_V, a behavior rather similar to the WT was observed, except for the persistence of faint patches remaining at the end of the cycle of compression/decompression. By contrast, an increase of the lateral pressure on membrane containing G_100_V leads to the formation of ‘jagged’ patches with many sharp angles, a mark of membrane rigidity. These changes persist upon decompression as observed with VAMP2VV.


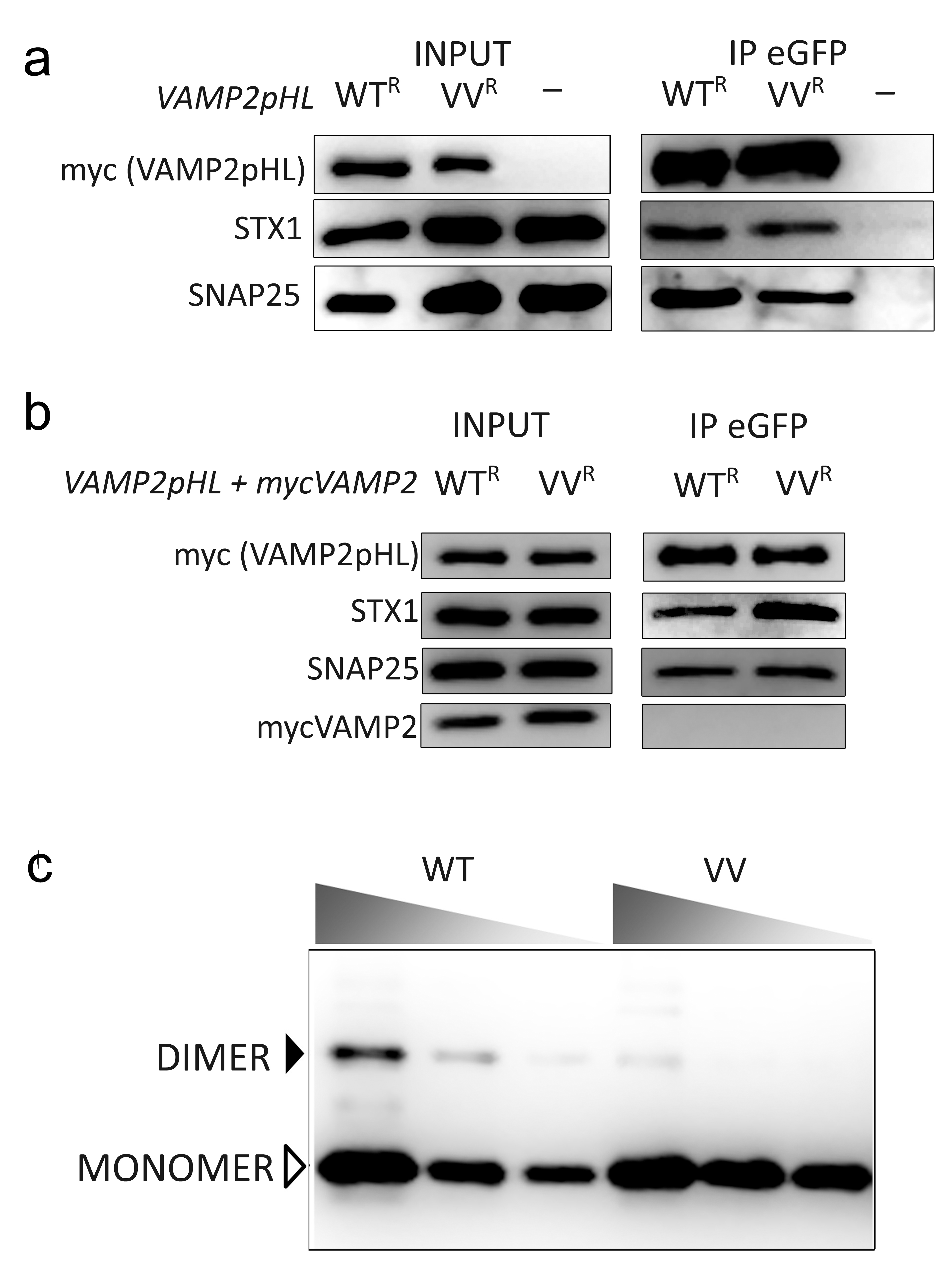


Supplementary Figure 4. **VAMP2 WT and VV mutant enter SNARE complexes and both form dimers**. (**a**) INS-1 832/13 cells were co-transfected with shVAMP2 and mycVAMP2pHL (wild-type [WT] or VV mutant). VAMP2pHL was immune-precipitated using beads coated an antibody anti-eGFP and samples were probed for the presence of mycVAMP2pHL using an anti-myc antibody or for the presence of endogenous syntaxin-1 (STX1) or SNAP25. For these analyses, 30% of the immune-precipitate and an aliquot corresponding to 3% of the total input to the immunoprecipitation reaction were applied to SDS-PAGE followed by immunoblotting. . It can be noted that the total amount of co-immuno-precipitated t-SNAREs was low, in line with the finding that only a small number of SNARE complexes exist in insulin-secreting cells prior to stimulation ^1^. (**b**) The presence of multimeric aggregates was tested by co-expressing mycGFP- and myc-tagged VAMP2 either as wild-type or as mutant proteins in INS-1 832/13 cells. Cells were co-transfected with shVAMP2 and equal amounts of mycVAMP2pHL and mycVAMP2 (both either wt or VV mutant). Immunoprecipitation and analysis of aliquots were performed as in A. Note the absence of interactions between mycVAMP2 and mycVAMP2pHL. The blot is representative for 3 separate experiments. Full blots are given in Supplemental Fig. 8. (**Cc**) Recombinant VAMP2 WT (left tracks) or VAMP2 VV (central tracks) were incubated and submitted to SDA-PAGE followed by immunoblotting using an anti-VAMP2 antibody. Decreasing concentrations of protein were used (50, 17 or 5 ng/µl). The blots a to c are representative for 3 separate experiments each. The VV mutant did not have an increased tendency to form dimers, in line with previously reported data ^2, 3^. Full blots are given in Supplemental Fig. 8. (**d**) Immunoblots of VAMP1 expression in rat brain (B), rat pheochromocytoma PC12 cells or rat clonal INS-1 832/13 β-cells. VAMP1 was detected by an isoform specific antibody and is present in brain homogenates (border lanes (B), 10 or 20 µg/lane) but absent in homogenates of PC12 cells (PC12, left lanes, 20 or 10 µg) and INS-1 832/13 cells (INS, right lanes, 20 or 10 µg).

**
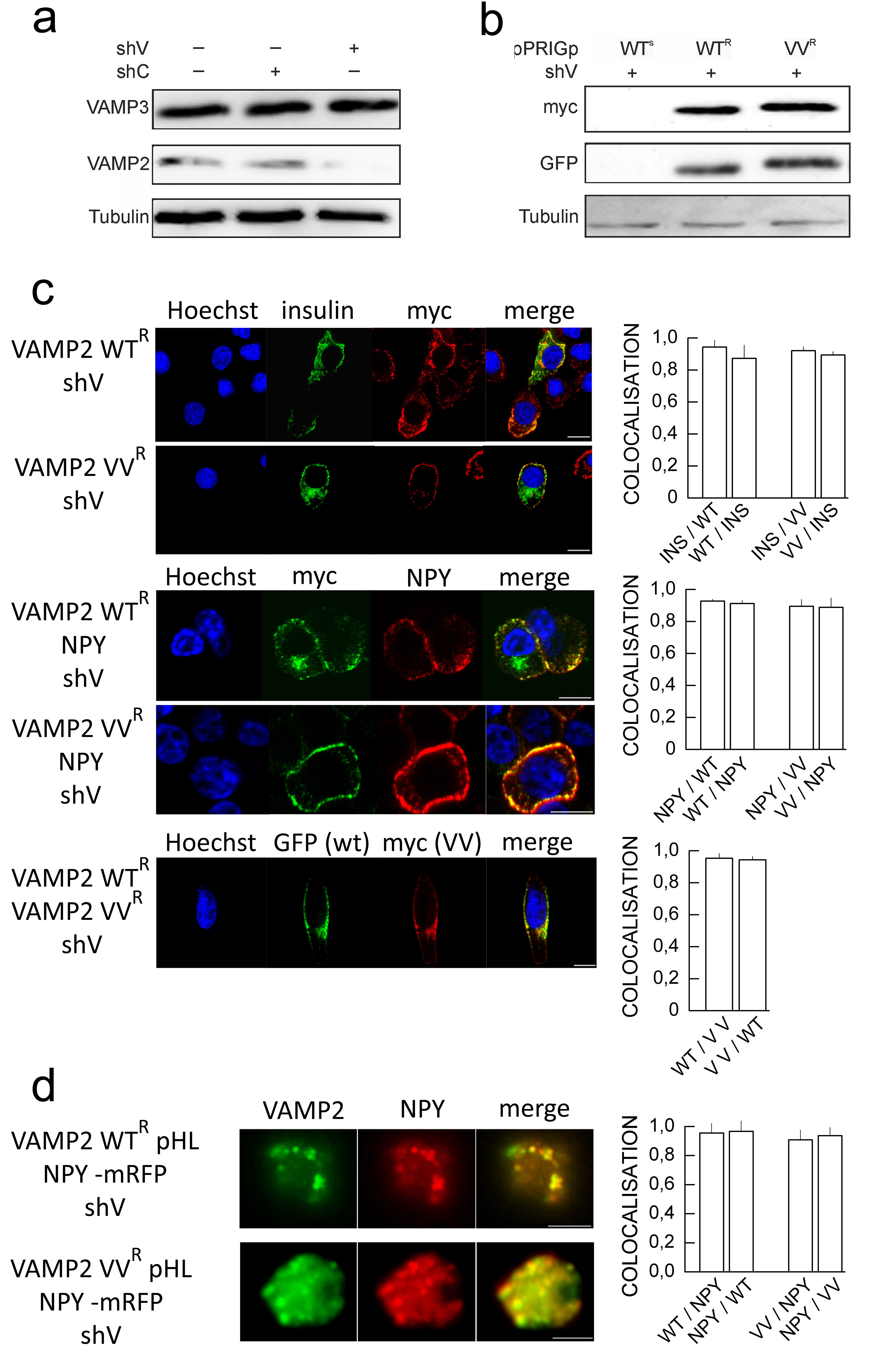
**

Supplementary Figure 5. **Knockdown of VAMP2 and re-expression of WT or mutant VAMP2 in INS-1 832/13 cells.**  (**a**) Transient expression of shV, but not shC, reduces expression of VAMP2 in INS-1 832/13 cells. Tubulin immunoreactivity (α-tub) is given as comparison. Note that the expression of VAMP3 is not altered. N=3, shown is a representative blot. (**b**) Re-expression of shRNA-resistant VAMP2 in INS-1 832/13 cells. Cells were co-transfected with a plasmid bearing shV, a bicistronic plasmid expressing eGFP and VAMP2. VAMP2 (myc signal) was either sensitive (wild-type, WT^S^) or resistant (WT^R^; VV^R^) to shV. (**c**) Subcellular localization of VAMP2 WT^R^ and VAMP2 VV^R^. Cells were co-transfected with shV and the indicated VAMP2 constructs and a plasmid coding for NPY-eGFP (3^rd^ and 4^th^ panel). In the bottom panel the phluorin-tagged form of VAMP2wt and the myc-tagged form of VAMP2 VV was used. Transiently expressed VAMP2 is detected via its N-terminal myc-tag and the large dense core vesicles with anti-insulin or via the fluorescence of NPY-eGFP. Colocalisation (Mander’s coefficient) was determined as in Fig. 2 on 14 cells (upper panel), 12 cells (middle panel) for each condition or 20 cells (lower panel). Scale bars: 10 μm. (**d**) TIRF microscopy imaging of INS-1 832/13 cells co-transfected with shV, NPY-mRFP and either VAMP2 WT pHL or VAMP2 VV Phl. Cells were imaged at 22° C in buffer without glucose or calcium added. Colocalisation (Mander’s coefficient) was determined on 8 cells for each condition. Scale bars: 10 μm.


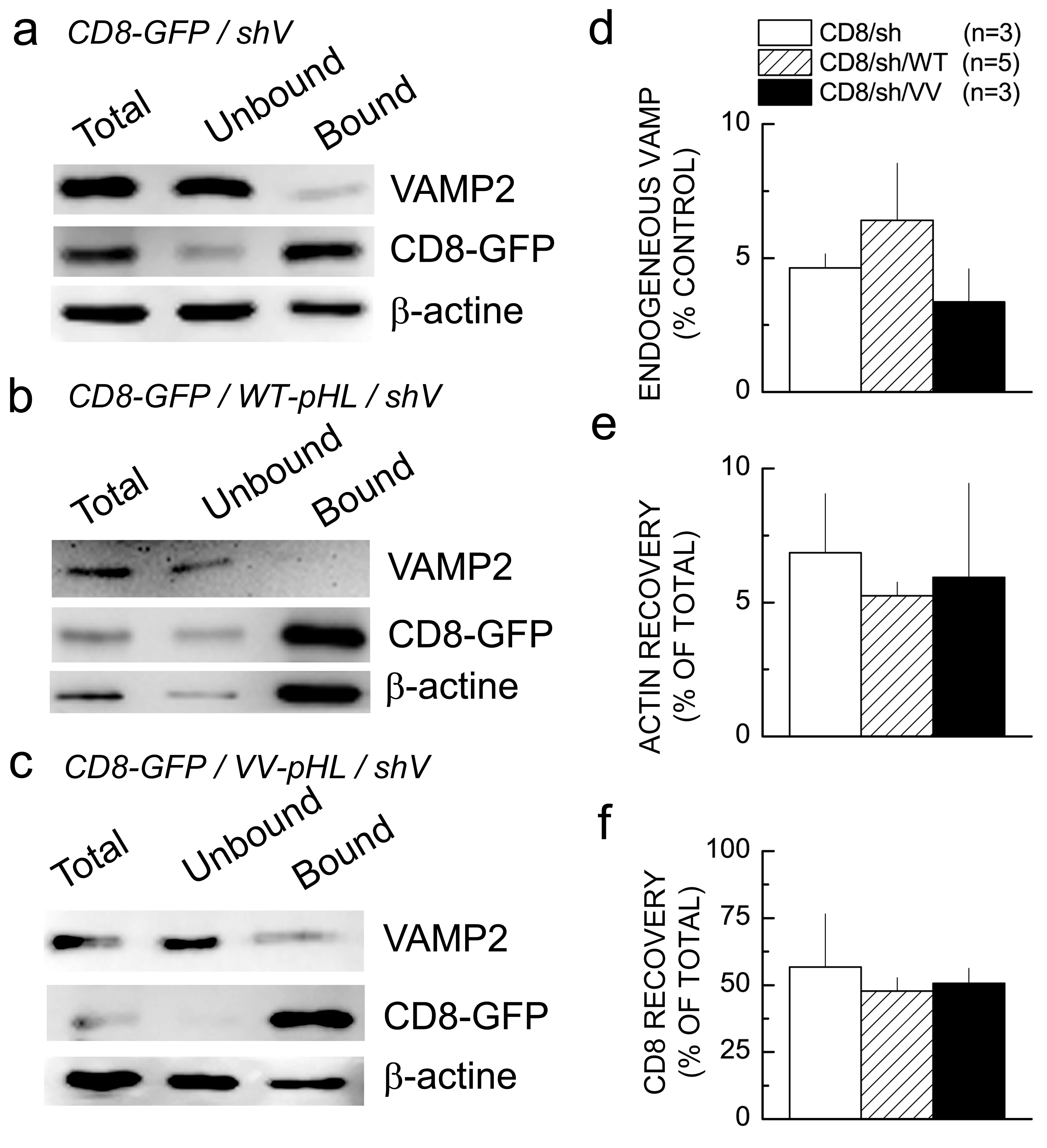


Supplementary Figure 6. **Quantification of endogenous VAMP2 in immuno-purified INS-1 832/13 cells after knockdown.** Transiently transfected cells were immuno-purified using a CD8-eGFP construct lacking the C-terminal intracellular catalytic domain and anti-CD8 Dynabeads ^4^. Cells were either transfected (**a**) with plasmids shV and CD8-GFP (replacing VAMP-pHL) to mimic conditions used in figures 5 to 7 or (**b, c**) triple transfected (with shV, CD8-GFP and VAMP-pHL WT or VV) to evaluate potential influence of VAMP WT or VV constructs on knock-down. Initial cell suspensions (total), unbound and bound fractions were analyzed by quantitative immunoblots and linearity as well as proportionality of the response was controlled by either standard curves using purified VAMP2-GST or serial dilutions of samples (100, 55 and 33%). 2.5% of total initial cell suspension, 3 % of unbound fraction and 50% of bound fraction were applied to SDS-PAGE and immunoblotted. (**d**) Quantification of residual endogenous VAMP2 levels in ummuno-purified cells. Integrated density values for immune-reactive bands of VAMP2 (total or bound) were normalized to corresponding actin bands and VAMP2 levels in immune-purified cells compared to expression levels of VAMP2 in the initial cell suspension. (**e and f**) percentage of recovered actin or CD8 immunoreactivity in immune-purified cells indicating that about half of CD8-GFP expressing cells were recovered corresponding to some 5% of cells in the initial cell suspension. Note that remaining endogenous VAMP2 may mainly reside on vesicles formed prior to transfection whereas the stimuli used in Figure 5-7 will preferentially fuse more recently formed vesicles, so called “newcomers”^6^. No statistical significant differences were observed in d-f using ANOVA, N= 3-5.


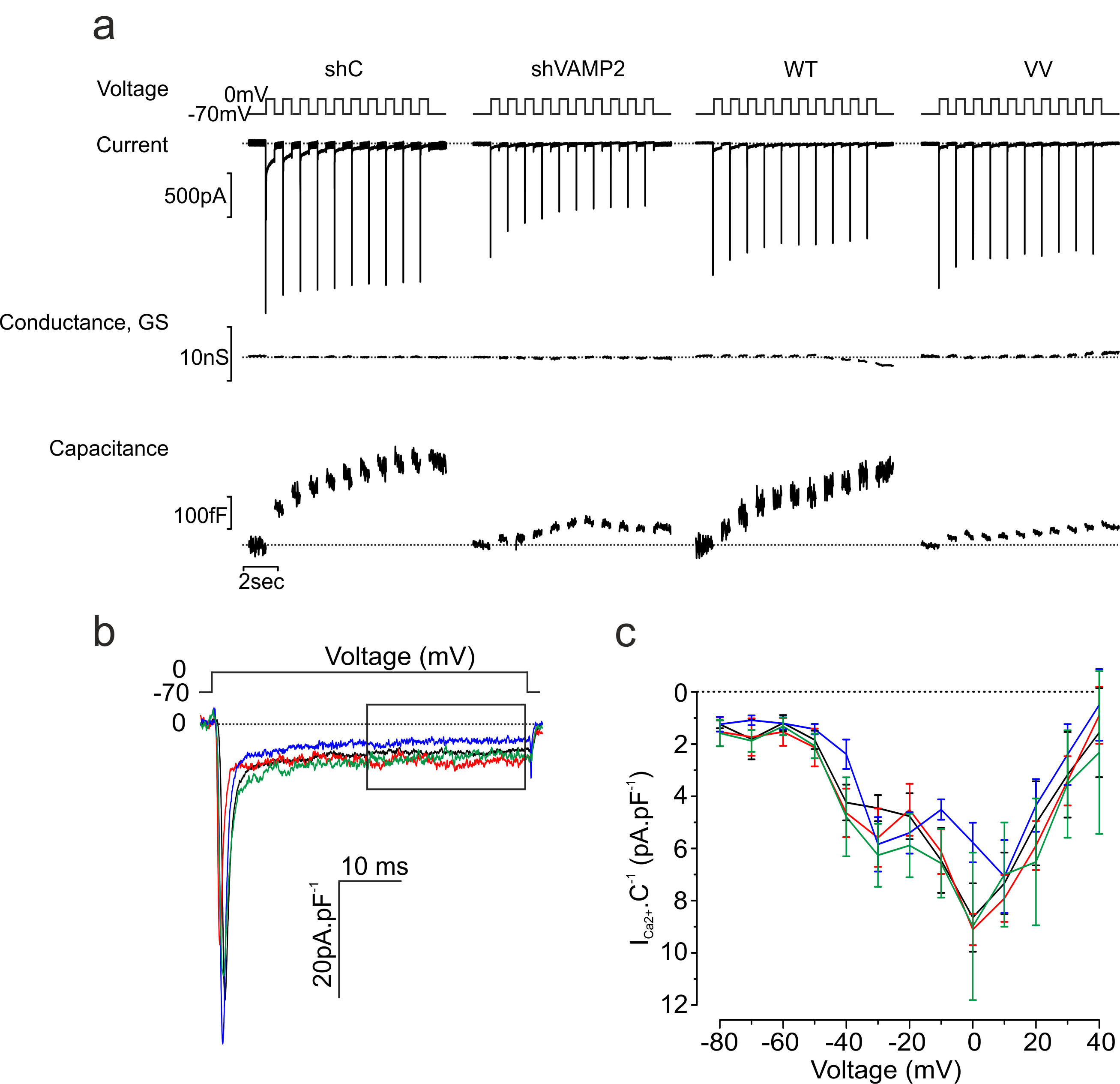


Supplementary Figure 7: **Raw measurements of membrane capacitance, and control of Ca^2+^ current density from INS1-832/13 transfected with VAMP2WT^R^ or VV^R^.** (**a)** Representative currents, conductance (GS), capacitance measurement elicited by a train of depolarizations from -70mV to 0mV. **(b)** Representative current elicited by a depolarization used above (higher resolution). The initial spiky component reflects activation of TTX-sensitive voltage-gated Na^+^ channels, whereas the sustained component represents the voltage-gated Ca^2+^ currents. We measured the amplitude of the sustained component during the period highlighted by the rectangle. **(c)** Current (I)-voltage (V) relationships recorded in control cells and after expression of the different constructs. To compensate for variations of cell size, Ca^2+^ channel activity is expressed as current density. Thus, the steady-state current amplitude (I) was normalized to cell capacitance (C; i.e. I/C). In all groups, the Ca^2+^-currents became detectable during depolarizations to -50 mV, displayed a shoulder between -40 and -20 mV with a secondary peak at ~0 mV and a voltage-dependent reduction at more positive voltages (reflecting the reduced driving force). The biphasic I-V relationship is a consequence of INS-1 832/13 cells, like primary rat β-cells ^5^, expressing both low- and high-voltage-gated Ca^2+^ channels. Importantly, there were no differences in the current densities between the groups.

Fig. 1d Fig. 1f


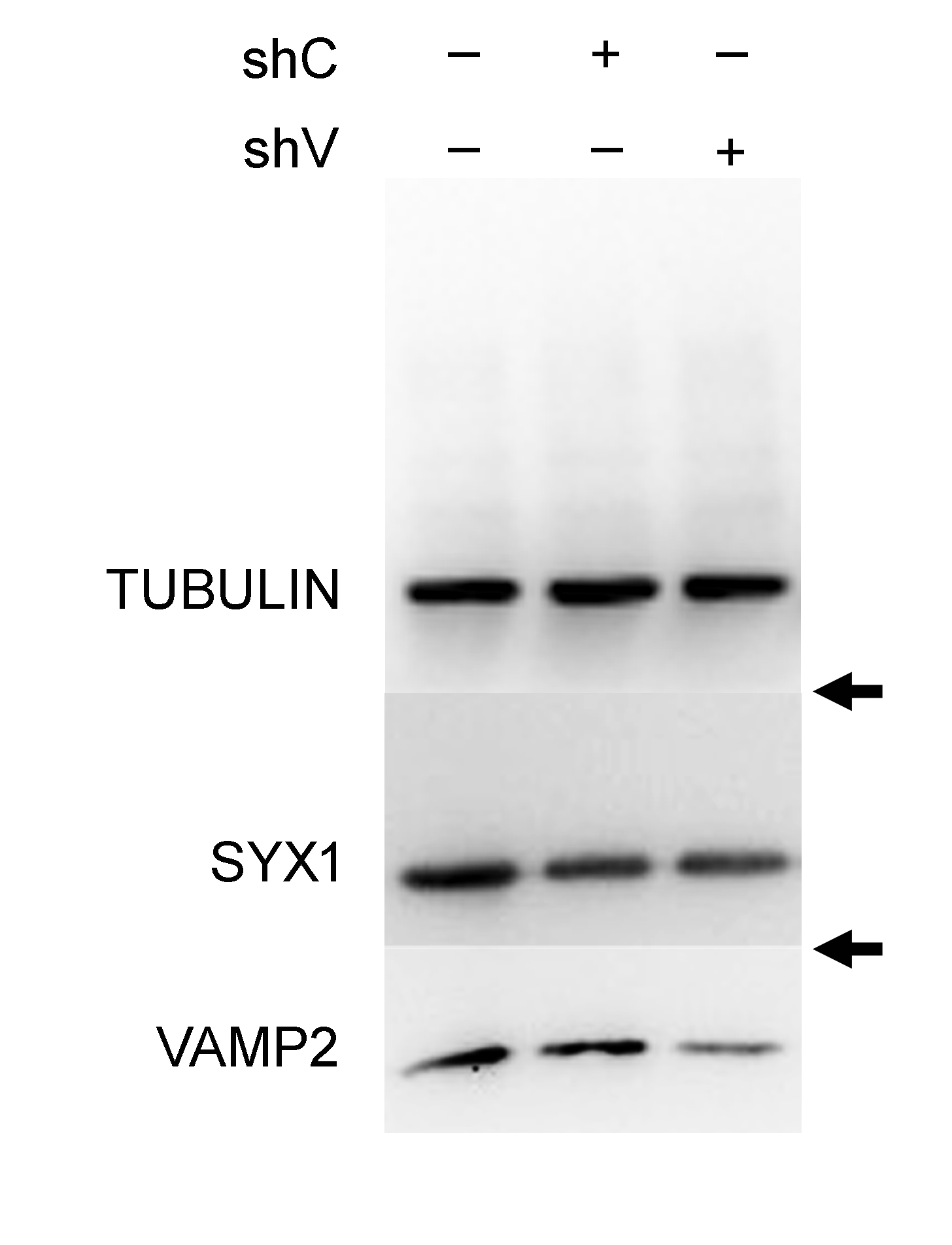


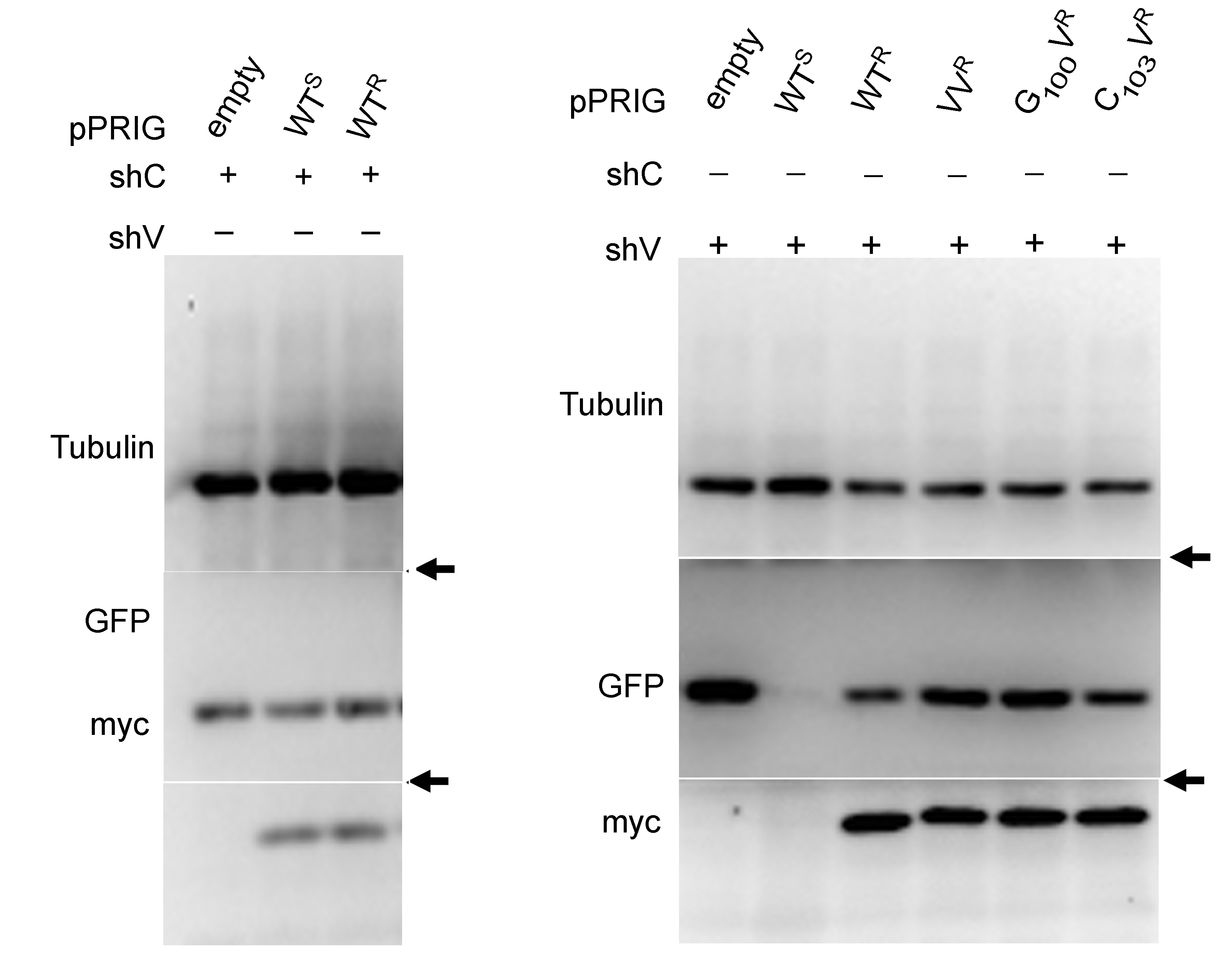


Suppl. Fig. 4a Suppl. Fig. 4b


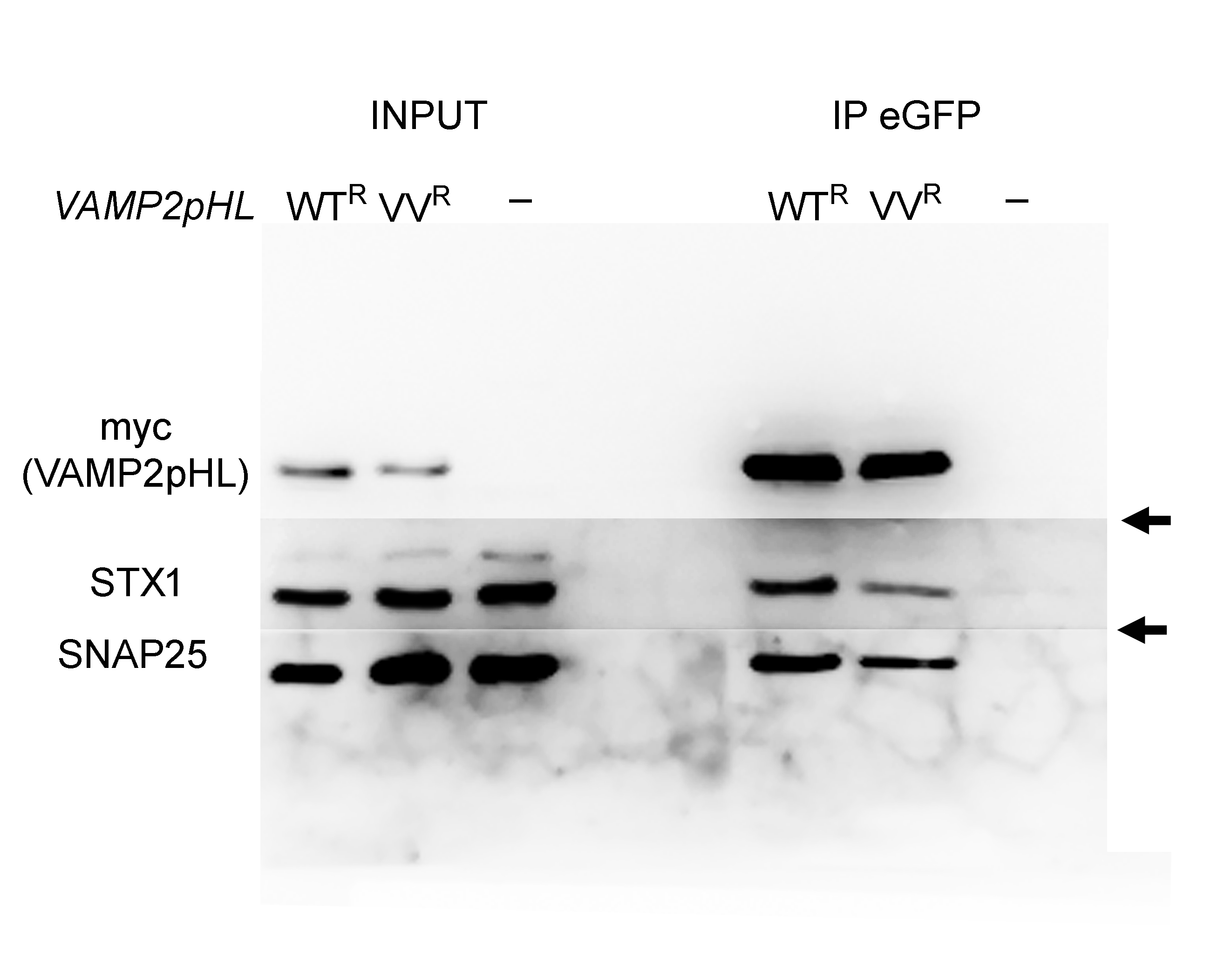


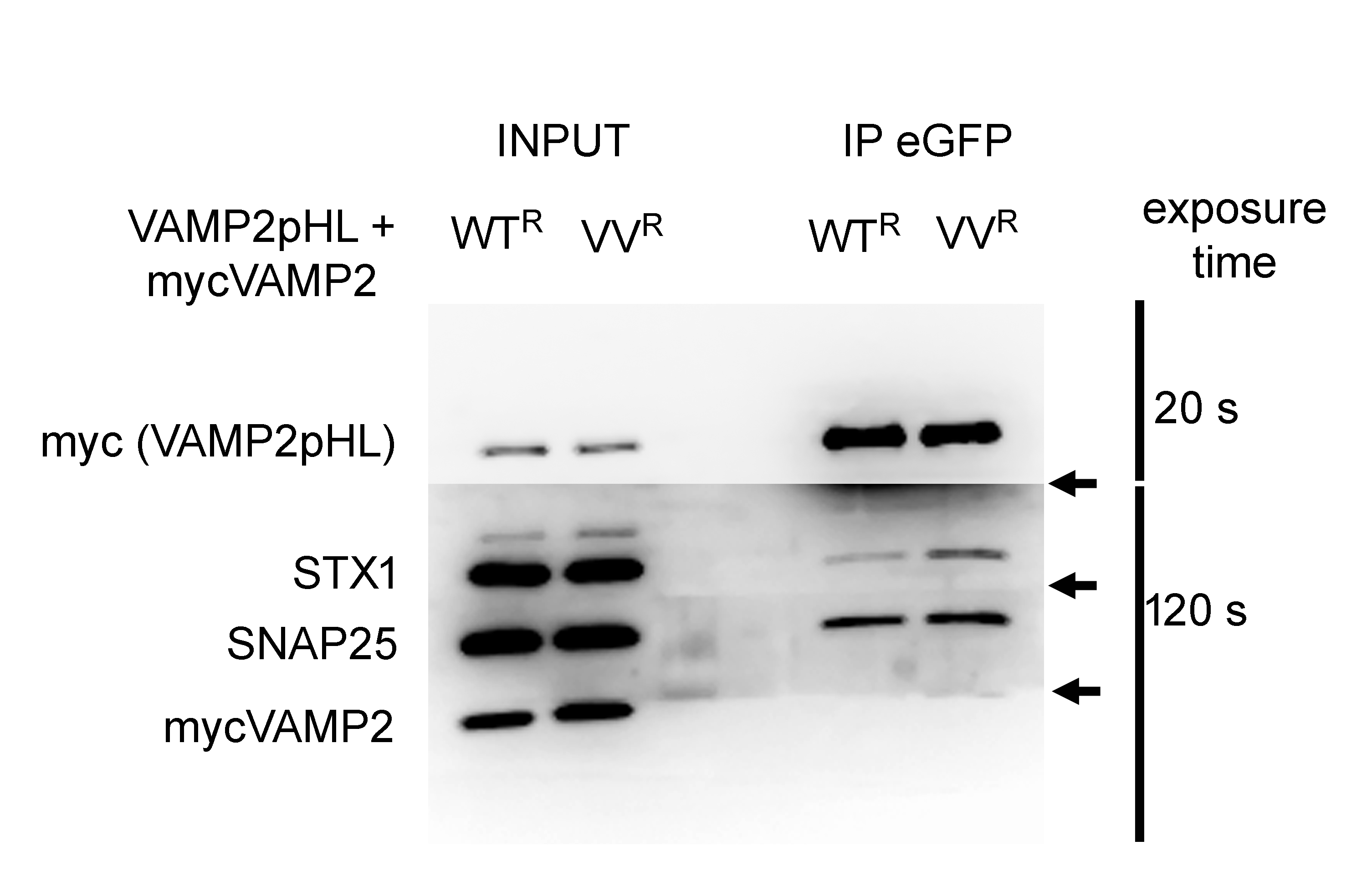


Suppl. Fig. 5a Suppl. Fig. 5b


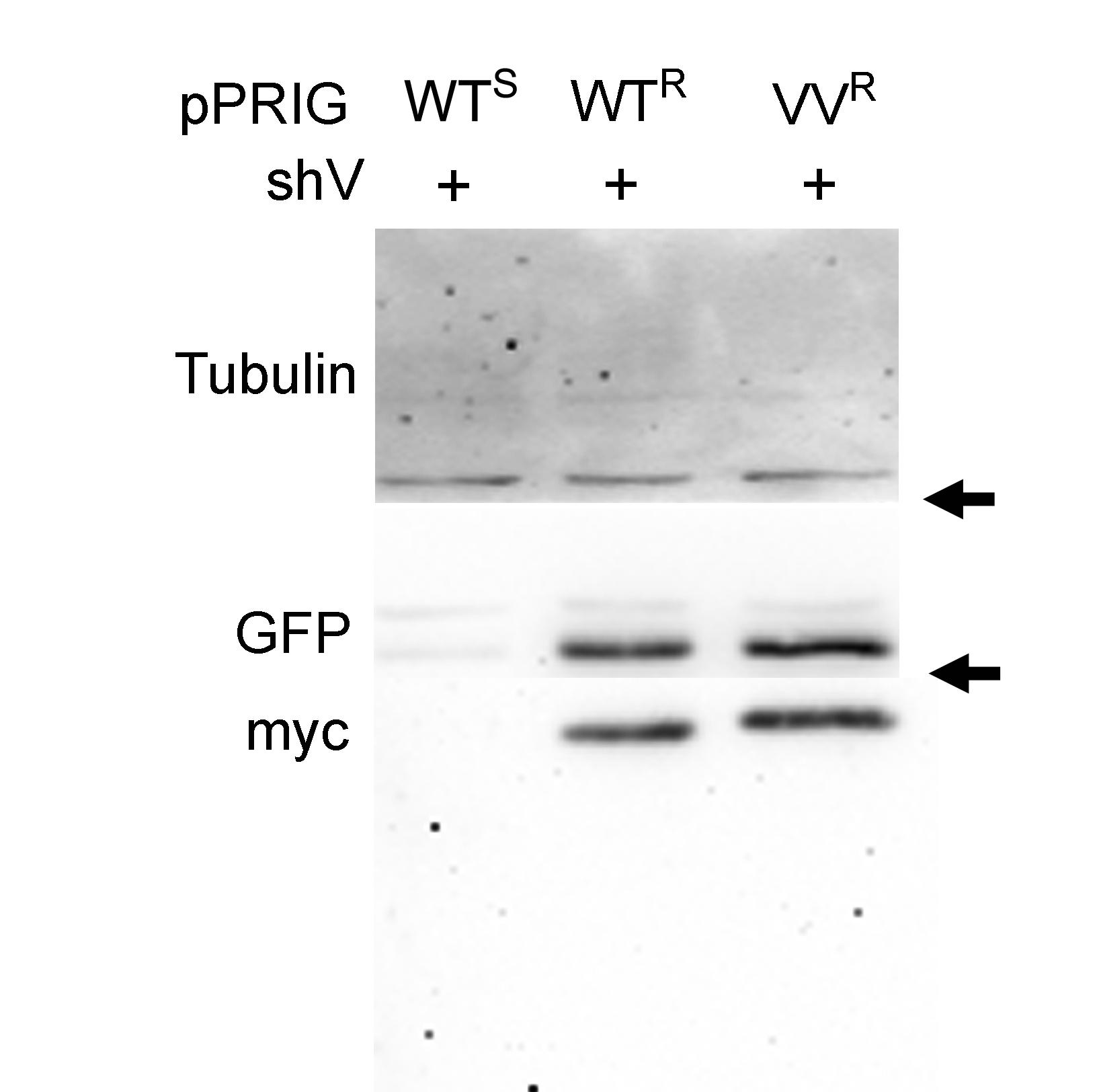


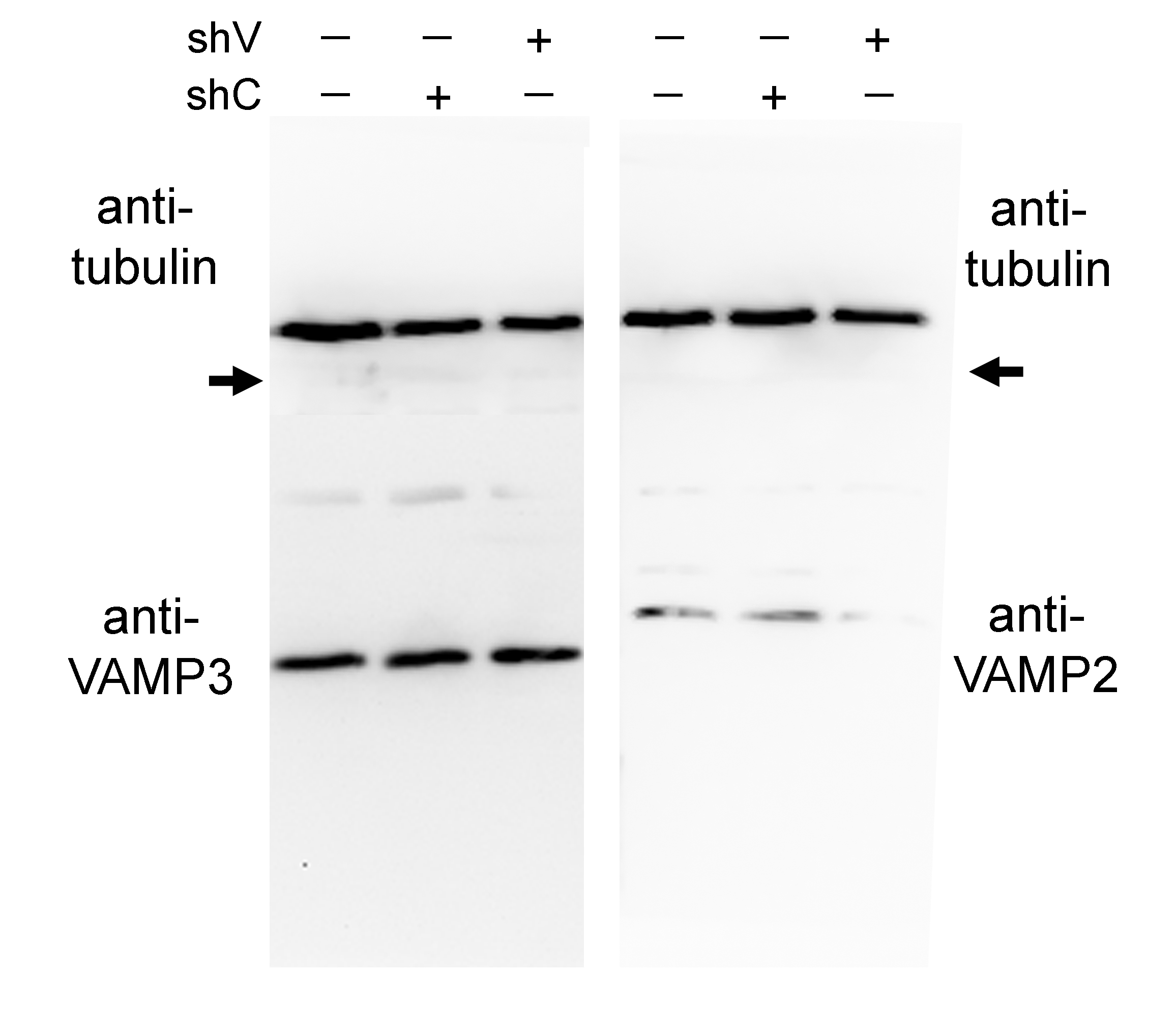


Supplementary Figure 8: **Full Immunoblots.** Note that blots were cut to permit incubation with distinct primary antibodies. Cuts are indicated by arrowheads. Panels are designated according to the original figure numbers and letters.

REFERENCES

1. Takahashi, N., W. Sawada, J. Noguchi, S. Watanabe, H. Ucar, A. Hayashi-Takagi, S. Yagishita, M. Ohno, H. Tokumaru, and H. Kasai, *Two-photon fluorescence lifetime imaging of primed SNARE complexes in presynaptic terminals and beta cells.* Nat Commun, **2015**. 6: p. 8531.

2. Fdez, E., M. Martinez-Salvador, M. Beard, P. Woodman, and S. Hilfiker, *Transmembrane-domain determinants for SNARE-mediated membrane fusion.* J Cell Sci, **2010**. 123: p. 2473-2480.

3. Laage, R. and D. Langosch, *Dimerization of the synaptic vesicle protein synaptobrevin (vesicle-associated membrane protein) II depends on specific residues within the transmembrane segment.* Eur J Biochem, **1997**. 249: p. 540-546.

4. Roger, B., J. Papin, P. Vacher, M. Raoux, A. Mulot, M. Dubois, J. Kerr-Conte, B.H. Voy, F. Pattou, G. Charpentier, J.C. Jonas, N. Moustaid-Moussa, and J. Lang, *Adenylyl cyclase 8 is central to glucagon-like peptide 1 signalling and effects of chronically elevated glucose in rat and human pancreatic beta cells.* Diabetologia, **2011**. 54: p. 390-402.

5. Ashcroft, F.M., R.P. Kelly, and P.A. Smith, *Two types of Ca channel in rat pancreatic beta-cells.* Pflugers Arch, **1990**. 415: p. 504-506.

6. Duncan, R.R., J. Greaves, U.K. Wiegand, I. Matskevich, G. Bodammer, D.K. Apps, M.J. Shipston, and R.H. Chow, *Functional and spatial segregation of secretory vesicle pools according to vesicle age.* Nature, **2003**. 422: p. 176-180.
